# Cocktail Therapy of Fosthiazate and Cupric-Ammoniun Complex for Citrus Huanglongbing

**DOI:** 10.3389/fpls.2021.643971

**Published:** 2021-03-31

**Authors:** Jingwei Duan, Xue Li, Junzhe Zhang, Baoping Cheng, Shuhan Liu, Hongmei Li, Quan Zhou, Wenli Chen

**Affiliations:** ^1^MOE Key Laboratory of Laser Life Science, Institute of Laser Life Science, College of Biophotonics, South China Normal University, Guangzhou, China; ^2^Guangdong Provincial Key Laboratory of Laser Life Science, College of Biophotonics, South China Normal University, Guangzhou, China; ^3^Guangzhou Key Laboratory of Spectral Analysis and Functional Probes, College of Biophotonics, South China Normal University, Guangzhou, China; ^4^Plant Protection Research Institute, Guangdong Academy of Agricultural Sciences/Guangdong Provincial Key Laboratory of High Technology for Plant Protection, Guangzhou, China; ^5^Department of Plant Pathology, Nanjing Agricultural University, Nanjing, China; ^6^Department of Radiology, The Third Affiliated Hospital of Southern Medical University, Guangzhou, China

**Keywords:** citrus Huanglongbing, fosthiazate, cupric-ammonium complex, cocktail therapy, transcriptome, synergistic effect

## Abstract

Huanglongbing (HLB) is a destructive citrus bacterial disease caused by *Candidatus* Liberibacter asiaticus (*Ca*.Las) and cannot be cured by current pesticides. Root lesion and *Tylenchulus semipenetrans* juveniles were observed in HLB-affected citrus tree roots. We hypothesize that root treatment with fosthiazate (FOS) and Cupric-Ammonium Complex (CAC) will improve the root growth and inhibit HLB. CAC is a broad spectrum fungicide and can promote growth of crops. FOS kills *Tylenchulus semipenetrans* and protects roots from damage by harmful bacteria such as *Ca*.Las. After 90 days of combination treatment of FOS and CAC through root drenches, the citrus grew new roots and its leaves changed their color to green. The inhibition rate of *Ca*.Las reached more than 90%. During treatment process, the chlorophyll content and the root vitality increased 396 and 151%, respectively, and starch accumulation decreased by 88%. Transmission electron microscopy (TEM) and plant tissue dyeing experiments showed that more irregular swollen starch granules existed in the chloroplast thylakoid system of the HLB-infected leaves. This is due to the blocking of their secretory tissue by starch. TEM and flow cytometry experiments *in vitro* showed the synergistic effects of FOS and CAC. A transcriptome analysis revealed that the treatment induced the differential expression of the genes which involved 103 metabolic pathways. These results suggested that the cocktail treatment of FOS and CAC may effectively kill various pathogens including *Ca*.Las on citrus root and thus effectively control HLB.

## Introduction

Huanglonging (HLB) is a major threat to citrus sustainable yield and production (Qureshi and Stansly, 2009). It is caused by the fastidious Gram-negative uncultivable bacterium belonging to the alpha-subdivision of the phylum Proteobacteria (*Candidatus* Liberibacter asiaticus; *Ca*.Las), the pathogen mainly colonizes the phloem tissue. They are transmitted through grafting, cuscuta (TuShiZi in Chinese), and insect vector (sap-sucking insects; *Diaphorina citri* in particular) in nature (Bove, 2006; Iftikhar et al., 2016). HLB can cause 30–100% yield losses. A tree will lose its fruiting capacity within 2–5 years from the date of symptoms appearance and its total life span will reduce to 7–10 years if no interventions/treatments are applied. HLB can threaten the profitability of fruit farmers with huge economic loss. Therefore, it is essential to develop effective and sustainable control strategies.

At present, the prevention and treatment of HLB mainly includes three areas: use of pathogen free nursery trees, control of psyllids to reduce the spread of HLB, and complete removal of HLB trees (Bassanezi et al., 2020). There are also various drug treatment methods, including the combination of benzbromarone and tolfenamic acid (Gardner et al., 2020), penicillin and streptomycin (PS) (Iftikhar et al., 2016), melatonin (Nehela and Killiny, 2020) and so on. There are also methods to reduce the contagion of HLB through management (Gottwald et al., 2007) and solar thermotherapy (Doud et al., 2017). The effective prevention of citrus HLB and alleviation of the plight of citrus industry has become a global problem. So far, there is no effective treatment for this disease (Li et al., 2019).

Normal plants tend to accumulate starch in leaves during the day and to consume these starch granules at night to maintain a dynamic balance. But for HLB-infected leaves, their chloroplasts hold large numbers of starch granules due to the interruption of metabolic pathways. High starch accumulation in leaves is a common HLB symptom (Guy, 1977), resulting in disintegration of the chloroplast thylakoid system and chlorosis symptoms in infected tissues (Schaffer et al., 1986).

Nematodes are the main cause of citrus slow decline disease. Among 40 nematodes species in citrus root, *Tylenchulus semipenetrans* is the most important cause of citrus slow decline disease (Song et al., 2016). *T. semipenetrans* causes citrus to lose between 10 and 30% of fruit yield. *T. semipenetrans* is a sedentary semi-endophytic parasite and affects all citrus species. It damages citrus root and makes plants susceptible to phytopathogenic fungi and/or bacteria, leading to serious illness (El-Borai et al., 2002). It has been previously noticed that nematode (*T. semipenetrans*) infestation is frequent in HLB-diseased citrus trees (Song et al., 2016). The commercial nematicide fosthiazate (FOS) is an organophosphorous pesticide and acts on acetylcholinesterase (Liao et al., 2019). Cupric-Ammonium Complex (CAC) such as Copper Dichlorotetramine or Sulfate-ammonia Complex and Cu-aminosulfate is a broad spectrum fungicide with low toxicity to humans and animals and has the function of promoting growth of crops (Li et al., 2016).

Based on the observed phenomena and our results, we hypothesize that FOS and CAC combined treatment will kill harmful bacteria in roots, improve the environment of soil, promote growth of new roots and leaves, and reduce starch content. Strong roots of citrus and healthy soil by the treatment of FOS and CAC will prevent invasion of pathogens including *Ca*.Las, as shown by our results that *Ca*.Las is gradually reduced during the 90 days of treatment.

## Materials and Methods

### Plant Material and Growing Conditions

#### Greenhouse Experiments

*Shatang Ju* trees (*Citrus reticulata Blanco cv. Shatang Ju*) were produced in our lab (113.35629°E, 23.143062°N) by grafting HLB-infected branches onto 2-year healthy *Shatang Ju* trees. These plants were then subsequently maintained in a greenhouse for 2 years. The experimental site has a subtropical monsoon climate with high temperatures, with an average annual temperature of 22.1°C. The annual average sunshine hours is 1,608 h, and the annual average relative humidity is 78%. On these plants, we conducted experiments of FOS and CAC combination treatment for 120 days, sampled leaves at different time points to quantitatively determine the content of HLB bacteria and observed the phenotype of the roots after treatment.

#### Orchard Experiments

A mildly HLB-infected orchard (8-year-old *Citrus reticulata Blanco cv. Shatang Ju)* was selected at Qiaotou Village in Shuangjiang Town of Dongyuan County, Heyuan City of Guangdong Province, China (114.704119°E, 23.945588°N). The area has a subtropical monsoon climate with high temperatures, with an average annual temperature of 21.0°C. The annual average sunshine hours are 1,687.0 h, and the annual average relative humidity is 77%. The annual average rainfall is 1,935 mm. The average annual rainfall in June is the highest, reaching 307 mm, and the least is December, with an average of only 36.5 mm. The average annual rainfall days are 154.3 days. The soil of the citrus orchard is mountain yellow soil with a soil depth of more than 60 cm. From this site, we took soil samples and photos of root phenotype. At the same time, we took corresponding leaves for quantitative determination of leaf HLB bacteria.

A severely HLB-infected orchard (8-year-old *Citrus reticulata Blanco cv. Shatang Ju*) was selected in Xiaoshan village, Conghua district, Guangzhou City, Guangdong Province, China (113.965218°E, 23.779132°N). The area has a subtropical monsoon climate with high temperatures, with an average annual temperature of 21.4°C. The annual average sunshine hours are 1,608 h, and the annual average relative humidity is 78%. The annual average rainfall is 1,950 mm. The average annual rainfall in June is the highest, reaching 310 mm, and the least is December, with an average of only 40 mm. The average annual rainfall days are 157 days. From this location, we took soil samples for *T.semipenetrans* juveniles identification, and corresponding leaf samples for quantification of *Ca*.Las.

### Treatments

#### Combination Treatment of FOS and CAC on Citrus Plants

The citrus trees were divided into group A and group B. Group A were treated with FOS and CAC combination therapy through root drenches, group B were treated with water as a control. The treatment method of group A is as follows: the roots of the citrus tree were first soaked in 1 L of Fosthiazate solution at a concentration of 480 μg/mL, applied as a soil drench (75% fosthiazate from Hebei Sannong Agricultural Chemical Co, Ltd.). After 3–5 days, we soaked 1L 200 μg/mL CAC to every citrus roots in greenhouse through root drenches (FOS or CAC dosage increased to 3 L for every tree in the orchard). Different leaf samples were collected at different days apart after post-treatment. The citrus orchards did not receive other phytosanitary treatments than those tested during the study period.

#### FOS or/and CAC Treatment on Bacteria for Antibacterial Effect Analysis

*Sinorhizobium meliloti* (*S. meliloti*) 1021 (kindly provided by professor Christian Staehelin) and *Agrobacterium tumefaciens* strain GV3101 (kindly provided by Dr. Jun Gu) were, respectively, cultured in TY solid medium and LB solid medium at 28°C for 2 days, then we inoculated a ring of bacteria with a loop of inoculum into 20 mL of TY liquid medium and LB liquid medium and incubated at 28°C and 180 rpm for 2 days. We picked a single clone and inoculated it into 5 mL medium. *S. meliloti* 1021 (antibiotic selection streptomycin 50 μg/mL) was shaken for 24 h, *Agrobacterium tumefaciens* strain GV3101 (antibiotic selection rifampicin 50 μg/mL) was shaken at 28°C for about 16 h. To measure bacteria growth to indicate bacteriostasis, we used the method of spectrophotometer turbidimetry. Take 1 mL of bacterial liquid and dilute it 20 times with TY or LB medium containing different concentrations of drugs (FOS and CAC), and a control group without adding drugs was set up in triplicate for each group. After shaking the culture in a shaker at 28°C and 180 rpm for 2 days, the bacterial suspension was measured using OD600 in a spectrophotometer. Our method of calculating antibacterial rate was:

$$\begin{array}{l} \text{the antibacterial rate }\%\;=\;\left(\text{O}{\text{D}}_{\text{A}}-\text{O}{\text{D}}_{\text{B}}\right)/\text{O}{\text{D}}_{\text{A}}\times 100\% \end{array}$$

(A represents the control group and B represents FOS or/and CAC treatment group). Each experiment was conducted in triplicate.

Transmission Electron Microscopy (TEM. Producer: Hitachi, Tokyo, Japan. Model: HT7700) experiments followed a previously published method (Liao et al., 2019).

Flow cytometry experiment was performed as previously described (Huang et al., 2019). The cells were then detected using a FACS Canto flow cytometer [Beckman Coulter Biotechnology (Suzhou) Co., Ltd.]. To evaluate the synergistic antibacterial effects of FOS and CAC, *S. meliloti* 1021 and *Agrobacterium tumefaciens* GV3101 were cultivated as described above in the presence of FOS, CAC or FOS + CAC combination. For *S. meliloti*, 400 μg/ml FOS and 267 μg/ml CAC were applied both singularly and in combination. For *A. tumefaciens*, the doses amounted to 160 μg/ml FOS and 133 μg/ml CAC. In both case, the combination treatments reach 100% antibacterial rate. One milliliter of culture was sampled for each antibacterial treatment. After antibacterial treatment, we collect the sample, centrifuged at 8,000 r/min for 5 min, discarded the supernatant, added 1×PBS buffer (pH7.4) to the precipitated bacteria, and added PI dye to a concentration of 10 μg/mL, incubate for 20 min at 4°C, then centrifuged at 4,505 g for 2 min, and wash twice with 1×PBS buffer. We used a flow cytometer (AccuriC6) FL2 channel [PI-labeled cells emit red fluorescence (FL2) at 546 nm] to determine the combining effect of FOS and CAC treatment on the cell membrane integrity of two Huanglongbing pathogen replacement bacteria *S. meliloti* 1021 and *Agrobacterium tumefaciens* GV3101.

### Plant Sampling

#### Root Sampling

##### For Identification of Nematodes

Six samples were randomly taken from 8-years-old *Shatang Ju* trees (*Citrus reticulata Blanco cv. Shatang Ju*) in orchards located at Xiaoshan village, Conghua district, Guangzhou City, Guangdong Province, China (113.965218°E, 23.779132°N). Procedures for sampling single-plant plots was as shown in Barker and Campbell (1981). About 1 kg rhizosphere soil with root tissues collected from ten spots under each tree were mixed as one sample to determine the presence of nematodes. At the same time, we took the leaves from the same tree to determine the HLB bacteria content.

##### For Transcriptome Analyses

The root samples for the transcriptome were derived from field citrus tree (*Citrus reticulata Blanco cv. Shatang Ju*) in Xiaoshan village, Conghua district, Guangzhou City, Guangdong Province, China (113.965218°E, 23.779132°N). Sample A: post-treatment root fiber (new roots), 90 days after FOS and CAC combination therapy; Sample B: pre-treatment root fiber (without Degraded Root tissue with Exposed Xylem, non-DREX); Sample C: pre-treatment root fiber (with Degraded Root tissue with Exposed Xylem, DREX). About three biological replicate samples (1 g each), each containing three root samples from different tree, were collected. Three replicates of three samples were sent to Beijing Novogene Technology Co, Ltd. for transcriptome analysis.

#### Leaf Sampling

*Shatang Ju* trees (*Citrus reticulata Blanco cv. Shatang Ju*) are derived from greenhouse samples in our lab (113.35629°E, 23.143062°N) (Graft HLB-infected branch to healthy 2-year-old *Shatang Ju* trees, grow in the greenhouse and use them after 2 years); slightly HLB-infected orchard (8-year-old *Citrus reticulata Blanco cv. Shatang Ju*) at Qiaotou Village in Shuangjiang Town of Dongyuan County, Heyuan City of Guangdong Province, China (114.704119°E, 23.945588°N); Severe HLB-infected orchard (8-year-old *Citrus reticulata Blanco cv. Shatang Ju*) in Xiaoshan village, Conghua district, Guangzhou City, Guangdong Province, China (113.965218°E, 23.779132°N). Since it is difficult to monitor root condition in field, we choose leaves as indicators of this treatment's success. We collect midrib of leaf sample at different time to measure the titers of *Ca*.Las. We just took a few leaves in the greenhouse. Out in the orchard, we randomly selected four directions and chose three leaves from each one. We collected them in an ice box, brought back to the lab, weighted the midribs and stored them at −80°C.

### Bacteria/Nematodes Detection and Identification

#### Identification of *Ca*.Las in Leaves

Leaf midrib samples, stored at −80°C, were rapidly ground in a mortar using liquid nitrogen. Total genomic DNA was extracted using Genomic DNA Geometry Extraction Kit of the Biotech Plant (Sangon, Shanghai, China) following the manufacturer's instructions. These genomic DNAs were used as templates for real-time qPCR to detect *Ca*.Las titer.

The recombinant plasmid pMD-19 with inserted 16S rDNA of HLB pathogen was obtained from the Guangdong Academy of Agricultural Sciences. Quantitative amplification of the recombinant plasmid pMD-19 were used (16S rDNA gene copies of HLB pathogen per gram plant tissue). The primers HLBas, HLBr, and HLBp (Zhou et al., 2011), specifically targeted at the 16S rDNA region of *Ca*.Las was used as a reference for the *Ca*.Las titer (Li W. B. et al., 2008). The pMD-19 plasmid was diluted into 10^−1^, 10^−2^, 10^−3^, 10^−4^, 10^−5^, 10^−6^, 10^−7^ as 7 templates for qPCR, and ddH_2_O was used as blank control. The DNA sample of healthy citrus leaves was a negative control and the experiment was repeated at least 3 times. All real-time qPCR amplifications were performed in an Applied BiosystemsTM QuantstudionTM 6 Flex.

The citrus roots were collected from 6.3 to 12.7 cm below the soil surface in four different quadrants within 0.6 m from the tree trunk, and then air-dried in a paper bag at room temperature (2–25°C) for about 24 h to facilitate easy removal of excessive soil by tapping with fingers (Park et al., 2018). Roots tissue was also immediately processed after collection or stored in a −80°C freezer.

For TaqMan real-time qPCR, the reaction mixture was performed in a total volume of 20 μl and contained 0.4 μl primers HLBas and HLBr each (Sangon, Shanghai, China), 0.4 μl probe HLBp (Takara, Liaoning, China), 10 μl TaqMan Mix (Takara, Liaoning, China), 1 μl DNA template, and 7.8 μl water. The real-time qPCR procedure is as follows: 95°C 5 min, 95°C 30 s, 60°C 30 s. Forty cycles. The calculation method of Li was used as a reference (Li et al., 2009).

#### Nematodes Extraction and Morphological Observation

Ten samples were randomly taken from 8-years-old *Shatang Ju* trees (*Citrus reticulata Blanco cv. Shatang Ju*) in orchards located at Qiaotou Village in Shuangjiang Town of Dongyuan County, Heyuan City of Guangdong Province, China (114.704119°E, 23.945588°N). Procedures for sampling single-plant plot was as shown in Barker and Campbell (1981). About 1 kg rhizosphere soil with root tissues collected from ten spots were mixed as one sample. At the same time corresponding to the rhizosphere soil samples, the leaf samples were taken to determine the quantitative HLB pathogen, as described in section Leaf Sampling.

Citrus roots were rinsed with tap water about 2–3 times, dried by tissue paper, and photographed by Canon EOS 60D.Nematodes were extracted from ca. 100 g mixture of soil and pieces of root samples from individual trees by the modified Baermann funnel (Viglierchio and Schmitt, 1983) for 24 h. Nearly 200–300 juveniles were recovered from soil extractable solution and the individuals were killed by heat and mounted on temporary slides. Morphology was observed on mounted specimens, light micrographs were produced using an Olympus BX51 microscope equipped with a camera.

#### Identification of *Tylenchulus semipenetrans* Juveniles in Roots

DNA was extracted from living nematode samples according to Li H. et al. (2008). The 28S D2-D3 region of ribosomal DNA (rDNA) was amplified with the forward primer D2A and the reverse primer D3B (De Ley et al., 1999). The primers were synthesized by Invitrogen, Shanghai, China. PCR conditions were as described by Li W. B. et al. (2008). PCR products were separated on 1% agarose gels and visualized by staining with ethidium bromide. PCR products of sufficiently high quality were purified for cloning and sequencing by Invitrogen, Shanghai, China. Contigs were assembled using Geneious R6.1.8. The resulting sequences were deposited in the GenBank with accession numbers MW051512 and MW051513.

### Molecular Analyses

#### Analyses on Nematodes

The obtained 28S sequences of *Tylenchulus semipenetrans* were compared with those of other Tylenchulus species available in GenBank using the BLAST homology search program. The selected sequences were aligned by MUSCLE (Edgar, 2004), which was followed by postalignment trimming with G-Blocks as implemented in SeaView Version 4 (Gouy et al., 2009). The GTR + I + G model was selected as the best-fit model of DNA evolution for 28S D2-D3 using jModelTest2 (Darriba et al., 2012) according the Akaike information criterion (AIC). The phylogenetic tree based on 28S rDNA sequences was obtained using MrBayes 3.2.6 (Ronquist and Huelsenbeck, 2003) with four chains (three heated and one cold). Model parameters were unlinked and the overall rate was allowed to vary across partitions. The number of generations for the total analysis was set to 6 × 10^6^, with the chain sampled every 1,000 generations and a burn-in value of 25%. The 50% majority rule phylogenetic consensus trees were visualized using FigTree v. 1.4.3 (Rambaut, 2016) and Inkscape (Bah, 2011).

#### Transcriptome Analyses on Citrus Plants

Total RNA was extracted from root samples by TRIzol Reagent (Invitrogen, Thermo Fisher Scientific, Shanghai, China). The quality of mRNA including purity, quantity and integrity was tested using Nanodrop, Qubit, and Agilent 2100. RNA was sequenced using Illumina HiSeq 1000 at Novogene in Beijing (https://en.novogene.com). Raw data were analyzed using Illumina Casava 1.8, and three types of sequence reads, reads with adapter, no exact base information, or low-quality reads, were filtered and removed. RNA libraries were constructed from ≥1 μg of total RNA. The kit used to build the library was Illumina's NEBNext® UltraTM RNA Library Prep Kit. On inserts of the expected size (library effective concentration is higher than 2 nM), qRT-PCR was performed and quantification was used to ensure library quality.

All raw-sequence reads data were uploaded in NCBI Sequence Read Archive (SRA, https://www.ncbi.nlm.nih.gov/sra/PRJNA680245) with accession number SRA414710.

The raw data obtained by sequencing contains a small number of reads with sequencing adapters or low sequencing quality, as shown in the Supplementary Table 3.

The clean reads filtered from the raw reads were mapped to the reference genome of C. reticulata (https://www.citrusgenomedb.org/organism/Citrus/reticulata).

DESeq software was used to analyze the differentially expressed genes (DEGs) using a negative binomial distribution *p*-value estimation model, with the differentially expressed gene screening standard set to padj <0.05. Other specific steps are included in Supplementary Figure 6.

We use clusterProfiler software to perform GO (Gene Ontology) function enrichment analysis on differential gene sets, KEGG (Kyoto Encyclopedia of Genes and Genomes) pathway enrichment analysis, etc. GO is a comprehensive database describing gene functions, which can be divided into three parts (biological process, cellular component and molecular function). GO function enrichment takes *p*-values < 0.05 as the threshold for significant enrichment. KEGG is a comprehensive database that integrates genomic, chemical, and system function information. KEGG pathway enrichment takes padj < 0.05 as the threshold of significant enrichment. A scatter diagram was used to display KEGG enrichment analysis results. The KEGG enrichment degree was measured based on the rich factor, Q value, and gene counts enriched in this pathway. Pathways with corrected *p*-values < 0.05 were determined to be significantly enriched in DEGs. As the number of enriched pathways counts was <20, all of them were plotted.

### Starch Accumulation Detection

Samples were taken between 2 and 4 p.m. from Fruit Tree Institute, Guangdong Academy of Agricultural Sciences (113.379079°E, 23.158071°N). Transmission Electron Microscopy (TEM. Producer: Hitachi, Tokyo, Japan. Model: HT7700) (TEM, producer: hitachi, Model: HT7800) experiments followed a previously published method (Achor et al., 2010; Folimonova and Achor, 2010). PAS and phenol yellows dyeing experiments were performed according to Brodersen et al. (2014). Fresh *Ca*.Las-infected and healthy leaves were fixed with formaldehyde, and sent to Servicebio Technology Co. Ltd. (Wuhan, China).

Starch accumulation was determined on severely infected samples from Xiaoshan village, as described in section Leaf Sampling. After washing in 80% ethanol, to separate starch from soluble sugars, starch was decomposed to glucose by acid hydrolisis, and colorimetrically quantified after reaction with anthrone. The Starch content detection kit [Soleibao Biotechnology Company for detection (BC0700)] was used for this purpose. Standard curve drawing: 0, 0.2, 0.1, 0.05, 0.025, 0.0125, 0.00625, 0.003125, 0.00156 mg/mL glucose standard solution as the abscissa and Absorbance at 620 nm (A_620_) as the ordinate to draw the standard curve:

$$\begin{array}{l} {\text{A}}_{620}=2.334x+0.1664 \end{array}$$

The goodness for fit of the standard curve *R*^2^ = 0.9892. Substitute A_620_ into the equation to get starch content x (mg/mL).

### Statistical Analysis

All tests were repeated at least three times and the results were analyzed by GraphPad Prism 6.0. Statistical analyses were performed using Dunnett's test (^*^p < 0.05, ^**^p < 0.01, and ^***^p < 0.001). Each value was the mean ± S.D. of three independent replicates. All statistical analyses were performed using the software spss statistics 23 with the significance level of 0.05.

## Results

### *Tylenchulus semipenetrans* Juveniles and Root Lesion Phenotype in HLB-Infected Citrus Trees

**Figure 1:**
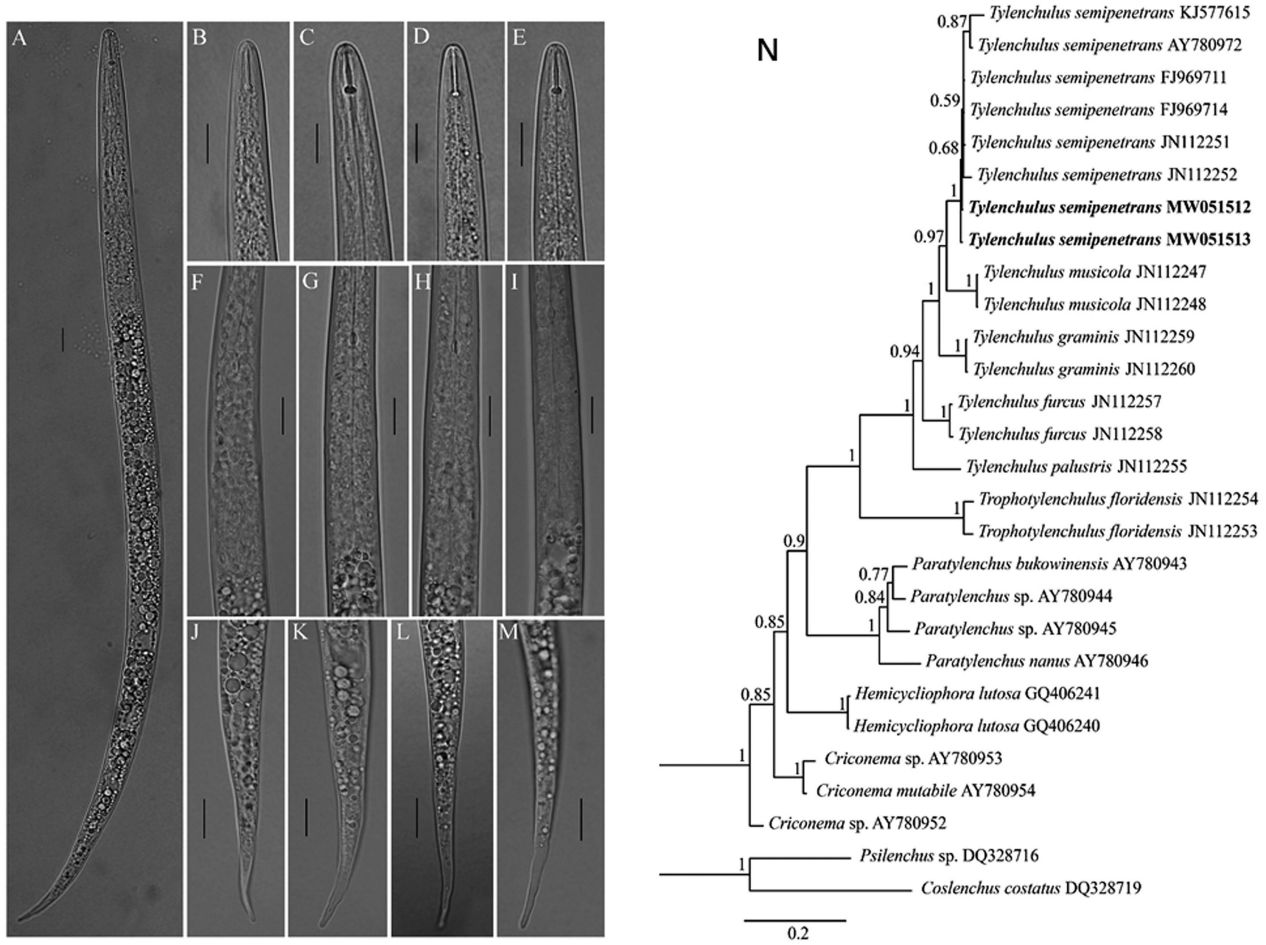
Light photomicrographs of *Tylenchulus semipenetrans* juveniles and Bayesian tree. **(A)** Entire body. **(B–E)** Anterior body region. **(F–I)** Esophageal glands; **(J–M)** Tail (Scale bars = 10 μm.). **(N)** Bayesian tree inferred from *Tylenchulus* spp. and other nematodes based on D2–D3 of 28S ribosomal DNA. Posterior probability values exceeding 50% are given on appropriate clades. New sequences original to this study are indicated in bold.

*Tylenchulus semipenetrans* juveniles were detected with severely HLB-infected “Shatangju” trees root (Supplementary Table 1). Light photomicrographs of *Tylenchulus semipenetrans* juveniles are shown in Figures 1A–M. Bayesian tree confirmed that it was indeed *Tylenchulus semipenetrans* (Figure 1N). It has been reported that citrus suffers from nematode damage (Song et al., 2016), which may be one of the reasons that cause more pathogenic bacteria to invade the root tissue and increase the HLB of the root.

**Figure 2:**
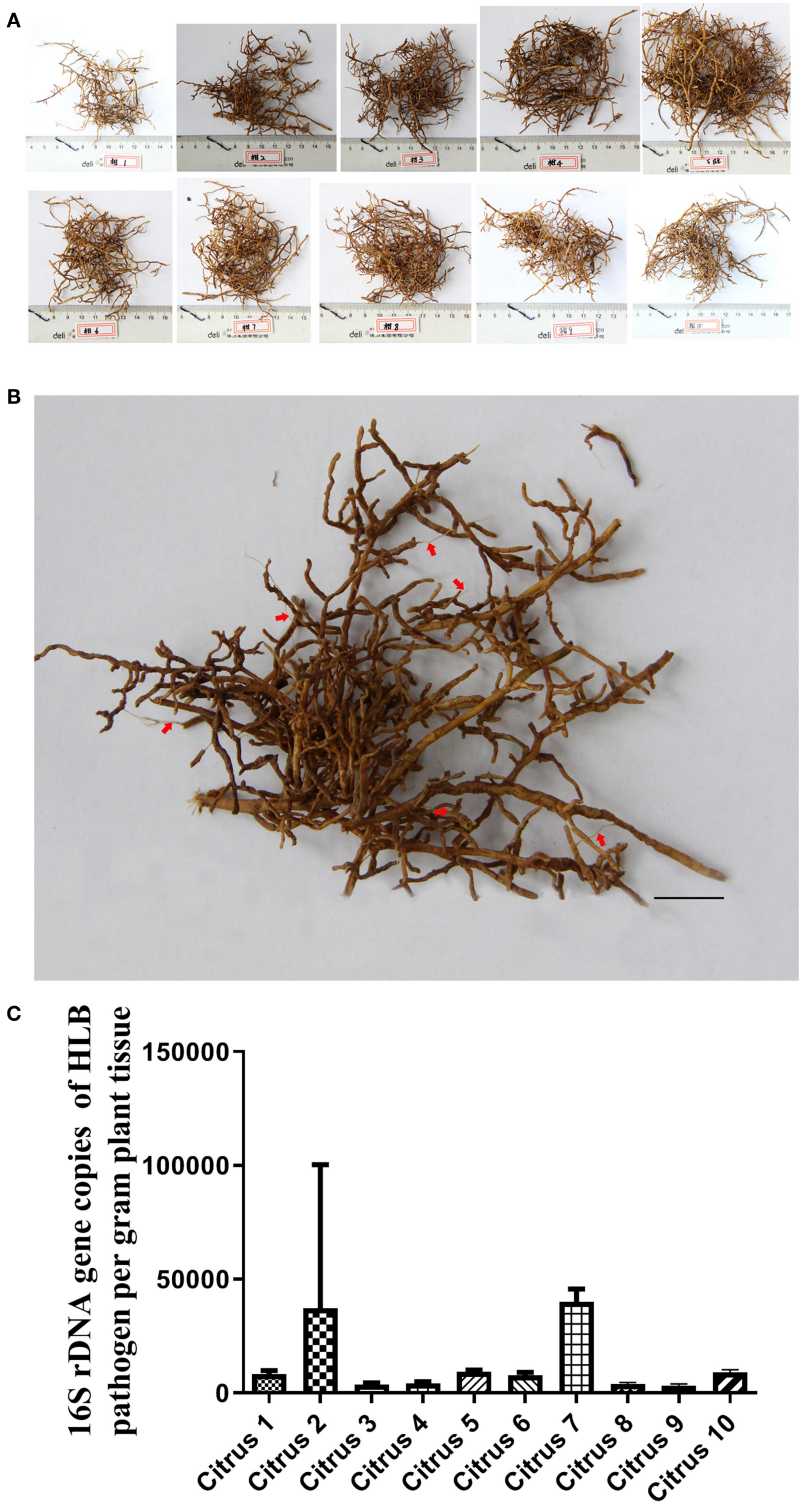
Root lesion phenotype in citrus trees with slight HLB infection. **(A)** visual appearance of mildly HLB-infected tree roots. **(B)** detail of one root system presented in **(A)**. Red arrows indicate lesions with tissue degradation and exposed xylem (Scale bars = 10 mm.) **(C)** HLB bacterial population in leaf midrib samples from plants corresponding to **(A)**. And the standard curve used is 16S rDNA gene copies of *Ca*.Las per gram leaves midrib corresponding to **(A)**, Y = −0.286X + 11.924, Y represents 16S rDNA gene copies of HLB pathogen, X represents Ct value. These data consistent with Supplementary Table 2.

Root lesions were found in mildly HLB-infected citrus trees (Supplementary Table 2 and Figure 2C). There were a lot of Degraded Root tissue with Exposed Xylem (DREX) in ten samples (Figure 2A). One example of DREX was pointed by arrows in red color (Figure 2B). DREX was also showed in severe HLB-infected “Shatangju” trees (Supplementary Figures 1C,D, DREX is labeled).

The appearance of leaves is a crucial symptom to detect whether the citrus tree is infected by HLB (Fleites et al., 2014), but we found that *Ca*.Las were more likely accumulated in DREX than non-DREX. Our results showed that the number of HLB pathogen is greatest in DREX, lower in non-DREX and lowest in HLB leaves (Supplementary Figure 2) on the same tree. We detected more *Ca*.Las in roots than in leaves. We originally sampled DREX materials for transcriptome analysis, but we found that most of DREX did not contain citrus RNA, as we could not obtain plant-derived genetic material from DREX. In fact, all the genetic material was from plant-associated organisms, including pathogens and pests (Supplementary Data 1), and we believe that *T. semipenetrans* infestation is enhanced by HLB disease, resulting in DREX and degradation of plant DNA. For this reason, we designed a combined treatment with FOS (commercial broad-spectrum efficacy for nematodes on citrus root) and CAC (a broad-spectrum fungicide) to treat citrus HLB and got significantly good results.

### Combination of FOS and CAC Effectively Relieved Citrus HLB

**Figure 3:**
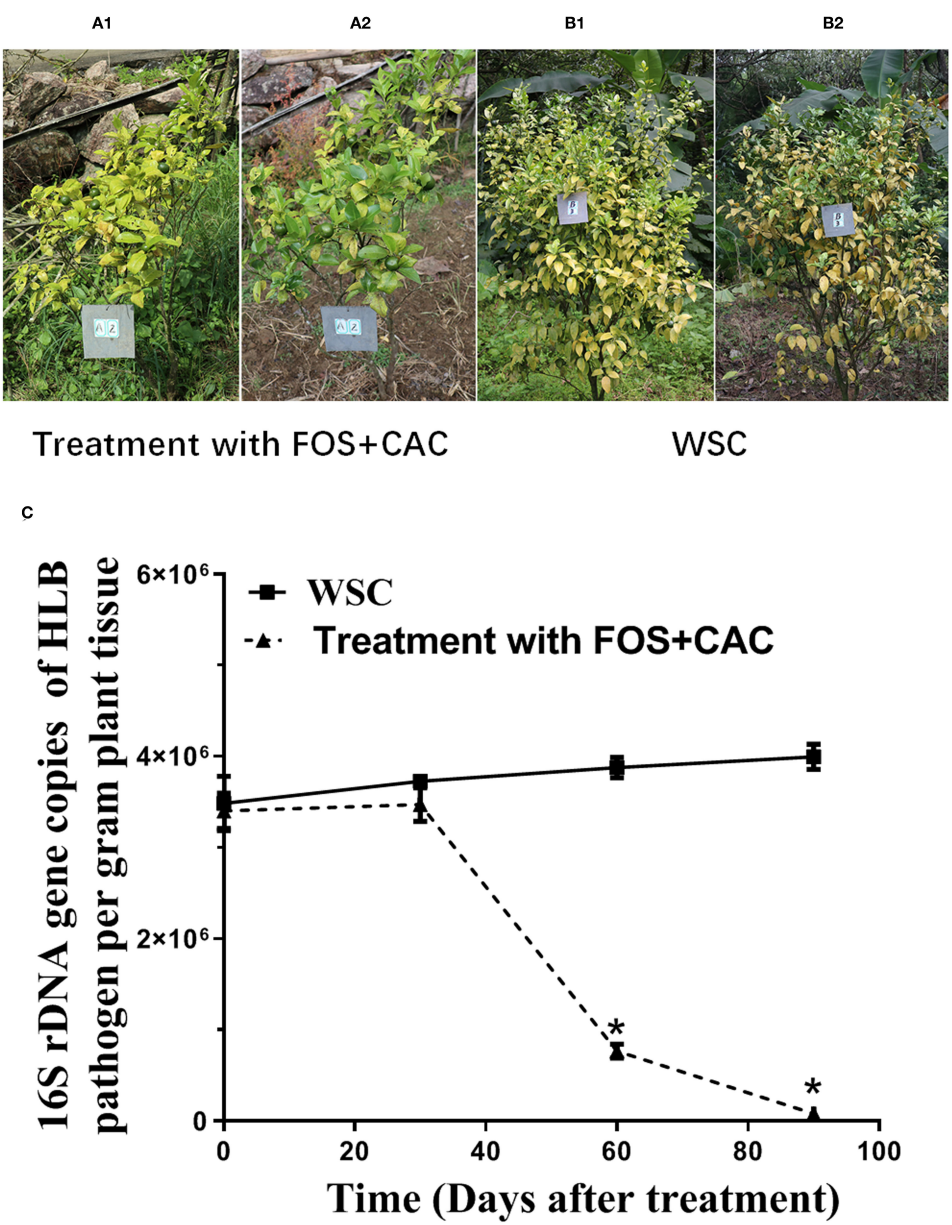
Combination of FOS and CAC effectively relieved citrus HLB after 3 months treatment in the orchard. **(A)** HLB-affected citrus after 3 months treatment. A1: before treatment; A2:3 months after FOS and CAC treatment. **(B)** HLB-affected citrus after 3-month without treatment. B1: before treatment; B2:3 months after water treatment. **(C)** 16S rDNA gene copies of *Ca*.Las per gram plant leaves midrib during 3 months in the orchard. Standard curve were obtained: Y = −0.310X + 10.927, *R*^2^ = 0.9965, Y represents 16S rDNA gene copies of HLB pathogen, X represents Ct value. WSC means water soak as control. An asterisk (*) indicates a significant difference (*P* < 0.05; Dunnett's test) between WSC and treatment with two drugs.

To confirm the practical usage of FOS and CAC, we conducted the test in an orchard field. Twelve HLB-infected citrus trees were equally divided into group A and group B. The orchard experiment showed that 60 days after treatment, the new leaves in group A trees apparently had normal green color (Figures 3A1,A2). This change didn't occur on group B trees (Figures 3B1,B2). The results showed that after the combined treatment of FOS and CAC combination, the number of *Ca*.Las of citrus leaves was significantly reduced after 90 days, while the control group was not (Figure 3C).

In order to further confirm the effectiveness of the cocktail of FOS and CAC for the HLB treatment, we conducted greenhouse experiments. We observed that the leaves of the HLB trees turned from yellow (Supplementary Figure 1A) to green (Supplementary Figure 1B) and a large number of new roots grew up, DREX is labeled (Supplementary Figure 1C). The number of *Ca*.Las in leaves were decreased 64% after treatment 30 days and further dropped 98% after treatment 90 days (Supplementary Figure 1D).

Obviously, the combined treatment of FOS and CAC lead to leaves turning green. We speculated that CAC itself can cause the greening of leaves and the improvement of the root environment in HLB-infected citrus. We confirmed that FOS and CAC combination treatment suppress the *Ca*.Las number of HLB-affected citrus 90 days after treatment.

### Synergistic Antibacterial Effect of FOS and CAC on Two Substitutes of *Ca*.Las

**Figure 4:**
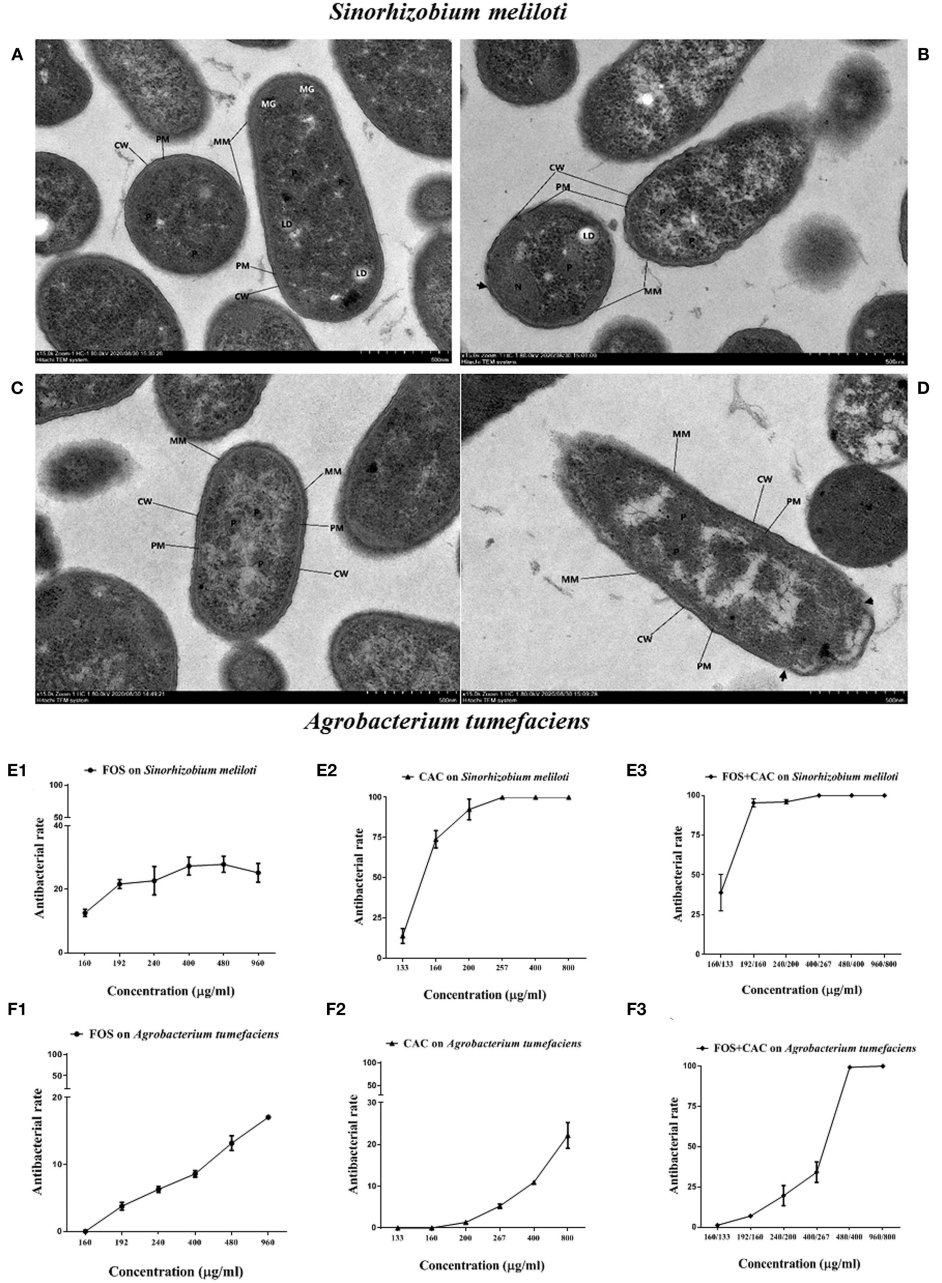
TEM images and antibacterial rate on two substitute bacteria of *Ca*.Las with FOS or/and CAC. **(A)** Control image of *Sinorhizobium meliloti*. **(B)** TEM image of *Sinorhizobium meliloti* with FOS + CAC (400/267 μg/mL) treatment. **(C)** Control image of *Agrobacterium tumefaciens*; the cell membrane (PM) is intact, no obvious plasmolysis and membrane breakage and dissolution are seen, and the periplasmic interval is normal. **(D)** TEM image of *Agrobacterium tumefaciens* with FOS + CAC (160/133 μg/mL) treatment; the protein mucosal layer (MM) is thin, and the local small area disappears, the cell wall (CW) showed irregular changes, partially broken or damaged (arrow). **(E)** Antibacterial rate of *Agrobacterium tumefaciens*. E1: FOS treatment alone; E2: CAC treatment alone; E3: FOS and CAC treatment. **(F)** Antibacterial rate of *Sinorhizobium meliloti*. F1: FOS treatment alone; F2: CAC treatment alone; F3: FOS and CAC treatment. Each group is divided into six concentration gradients, each gradient is repeated three times.

Since *Ca*.Las has not been successfully cultured *in vitro* so far, we used two bacteria *Sinorhizobium meliloti* and *Agrobacterium tumefaciens* as substitutes for the antibacterial experiments due to their close lineage (Stover et al., 2013; Hu et al., 2016), and tested the bactericidal effect of FOS and CAC drugs on them. Comparing with control (Figures 4A,C), TEM (Transmission Electron Microscopy) images of *S. meliloti* and *Agrobacterium tumefaciens* with FOS + CAC treatment showed that the mucosal layer (MM) was thin, local small area disappeared, cell wall (CW) showed irregular changes, partially broken or damaged (Figures 4B,D).

**Figure 5:**
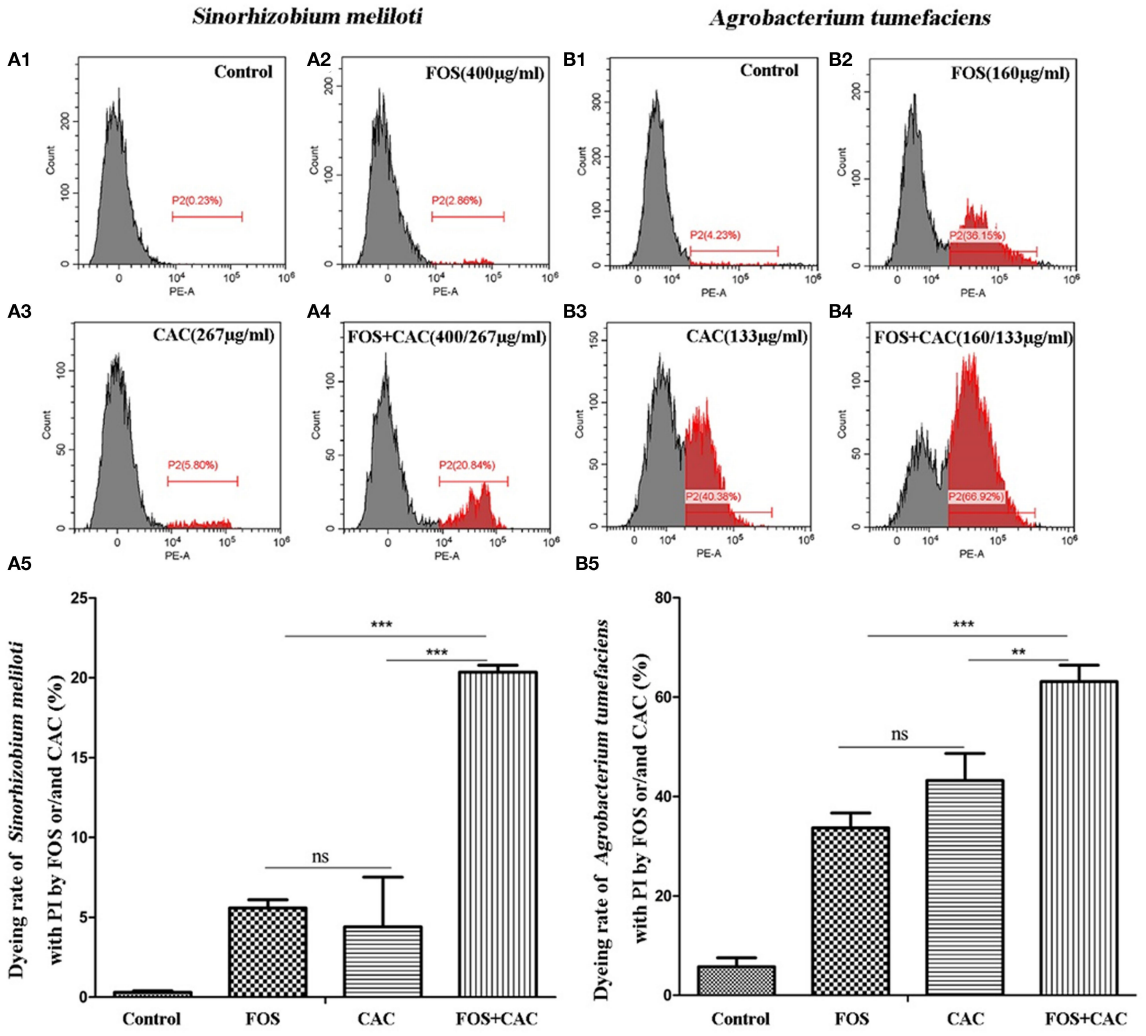
Flow cytometry to determine cell membrane permeability of FOS or/and CAC on *Sinorhizobium meliloti* and *Agrobacterium tumefaciens*. **(A)** Changes of cell membrane permeability of *Sinorhizobium meliloti* with FOS and/or CAC treatment. A1: control; A2: FOS (400 μg/mL); A3: CAC (267 μg/mL); A4: FOS (400 μg/mL) +CAC (267 μg/mL); A5: a statistical chart of A1, A2, A3, A4. **(B)** Changes of cell membrane permeability of *Agrobacterium tumefaciens* with FOS and/or CAC treatment. B1: control; B2: FOS (160 μg/mL); B3: CAC (133 μg/mL); B4: FOS (160 μg/mL) +CAC (133 μg/mL); B5: a statistical chart of B1, B2, B3, B4. Student's *t*-test, *p < 0.05, **p < 0.01, ***p < 0.001, * indicated significant differences, ns indicated no significant differences.

We found that CAC at 100–200 μg/mL effectively inhibited the growth of *S. meliloti*. The antibacterial rate of *S. meliloti* reached 70% at 200 μg/mL CAC and 100% at 267 μg/mL or higher CAC concentration (Figure 4E2). In contrast, FOS had a weak antibacterial effect on *S. meliloti* (Figure 4E1). The antibacterial rate of *S. meliloti* reached 100% when FOS 192 μg/mL + CAC 160 μg/mL were used together (Figure 4E3). As for *Agrobacterium tumefaciens*, the antibacterial rate of the two drugs is 10.93% (CAC at 400 μg/mL) and 13.16%, respectively, (FOS at 480 μg/mL) (Figures 4F1,F2), but when FOS 480 μg/mL + CAC 400 μg/mL were used in combination, their antibacterial rate reached 100% (Figure 4F3). We used flow cytometry to determine the permeability of FOS or/and CAC to the bacteria cells of *S. meliloti* and *Agrobacterium tumefaciens*, respectively. The results showed that the cell permeability caused by FOS + CAC is greater than that cause by FOS or CAC alone (Figure 5).

These results suggested that FOS may has a synergistic effect on CAC antimicrobial effect.

### The Changes of HLB-Infected Citrus Leaves' Starch, Total Chlorophll Content, and Roots Vigor Under FOS and CAC Combination Treatment

**Figure 6:**
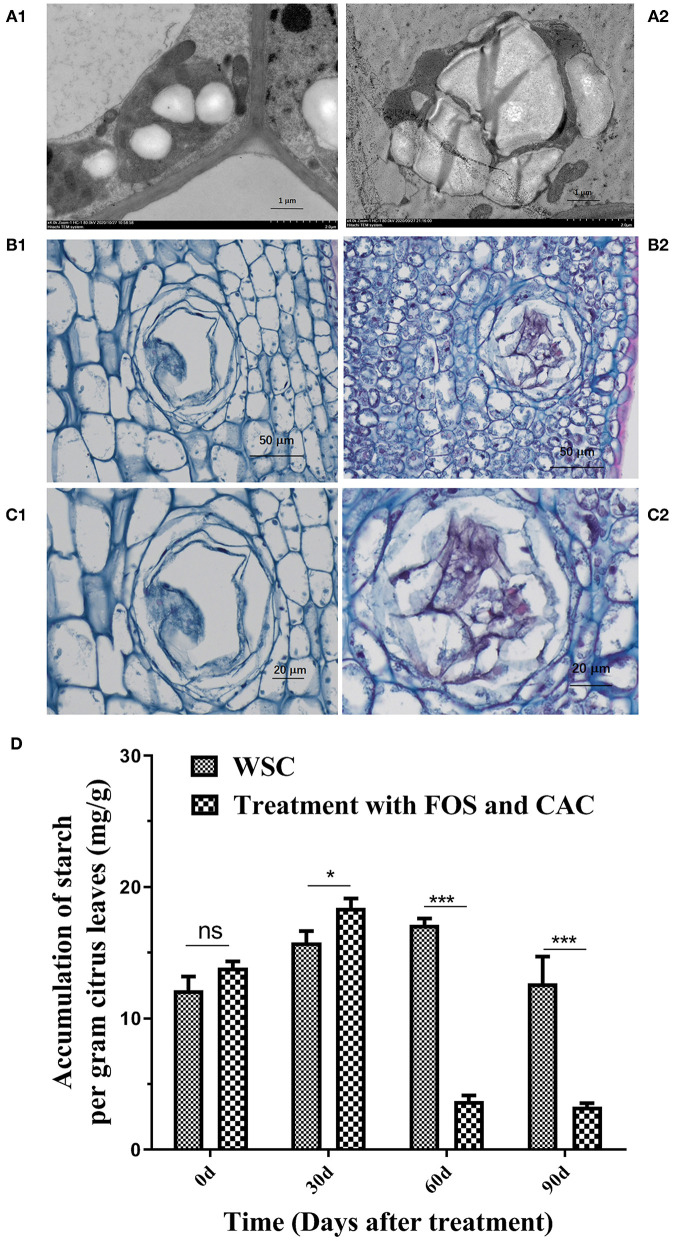
Starch accumulation on HLB-infected citrus leave and was reduced after FOS and CAC treatment. **(A)** TEM images of citrus leaves. A1: healthy leaves; A2: HLB-infected leaves. **(B)** PAS and phenol yellows dyeing of citrus leaves. B1: healthy leaves; B2: HLB-infected leaves. **(C)** The secretory tissues of citrus leaves in partial enlargement of **(B)**. C1: healthy leaves; C2: HLB-infected leaves. **(D)** The changes of starch accumulation during FOS + CAC combination treatment 90 days. Asterisk indicates significant differences during FOS + CAC combination treatment 90 days (Student's *t*-test, *p < 0.05, ***p < 0.001), ns indicated no significant differences.

TEM images of HLB-infected leaves showed more irregular swollen starch granules in chloroplasts compared to healthy leaves (Figure 6A1 and Supplementary Figure 3), which severely damaged the thylakoid structure in the chloroplast (Figure 6A2 and Supplementary Figure 3). PAS and phenol yellows dyeing showed that dyed light red granules in the parenchyma cells were all starch in HLB-infected leave (Figure 6B2). There was also a lot of starch accumulation in the secretory tissues of citrus leaves (Figure 6C2). Under normal circumstances, secretory tissues store aromatic volatile substances. In healthy plant leaves, these substances dissolve and volatilize during filming, so we see the cavity of secretory tissue (Figures 6B1,C1), but it is filled in HLB-infected leaves with many other substances (including starch). After the combined treatment of FOS and CAC, the starch content was reduced by nearly 88% on average after 60 days of treatment (Figure 6D), which indicated that FOS and CAC combined treatment reduced the starch accumulation in HLB-infected citrus leaves.

The total chlorophyll content was increased on average 396% after 90 days of treatment, while the control group gradually decreased (Supplementary Figure 4A). This was consistent with the results of the leaves turning green after treatment (Figure 3A2 and Supplementary Figure 1B). Roots vigor was increased on average 151% after 60 days of treatment (Supplementary Figure 4B), this is consistent with the results of new roots growth after FOS and CAC combination treatment (Supplementary Figure 1C). FOS and CAC treatment greatly enhanced *PR* (pathogenesis-related gene)*1* and *PR2* gene expression, when compared with using water only (Supplementary Figures 4C,D), suggesting that combined treatment of FOS and CAC increased the expression of disease resistance genes.

In order to better understand the HLB treatment effectiveness and screen out key genes, we sent the root samples (new roots with fiber, because a lot of new roots grew after the combined treatment) of post-treatment and Degraded Root tissue with Exposed Xylem (DREX) and HLB root fiber (Non-DREX) of pre-treatment to Beijing Novogene Technology Co, Ltd for transcriptome experiments. Among them, three samples of DREX were rotten roots. After RNA extraction and retrotranscription, low homology of cDNA to citrus sequences indicated that it was impossible to use the citrus database to further analyze the transcriptome. From the results of unmapped reads (Supplementary Data 1), we found that there were various pathogens genes in DREX and Non-DREX.

### Transcriptome Data Analysis Before and After Treatment

Total RNA was extracted with Illumina RNAseq, from which six cDNA libraries were constructed. The percentage of high-quality reads in each library was more than 92%. Afterwards, at least 72.5% of the total clean reads were mapped to the reference genome using the HISAT2 software. These results indicated the lack of contamination and that the reference genome was appropriately chosen (Supplementary Table 3). In this study, *R*^2^ (the square of the Pearson coefficient) was higher than 0.6 for both tested samples (Supplementary Figure 5), demonstrating the reliability of the experiments which could reveal different gene expression between samples (Qiu et al., 2020).

**Figure 7:**
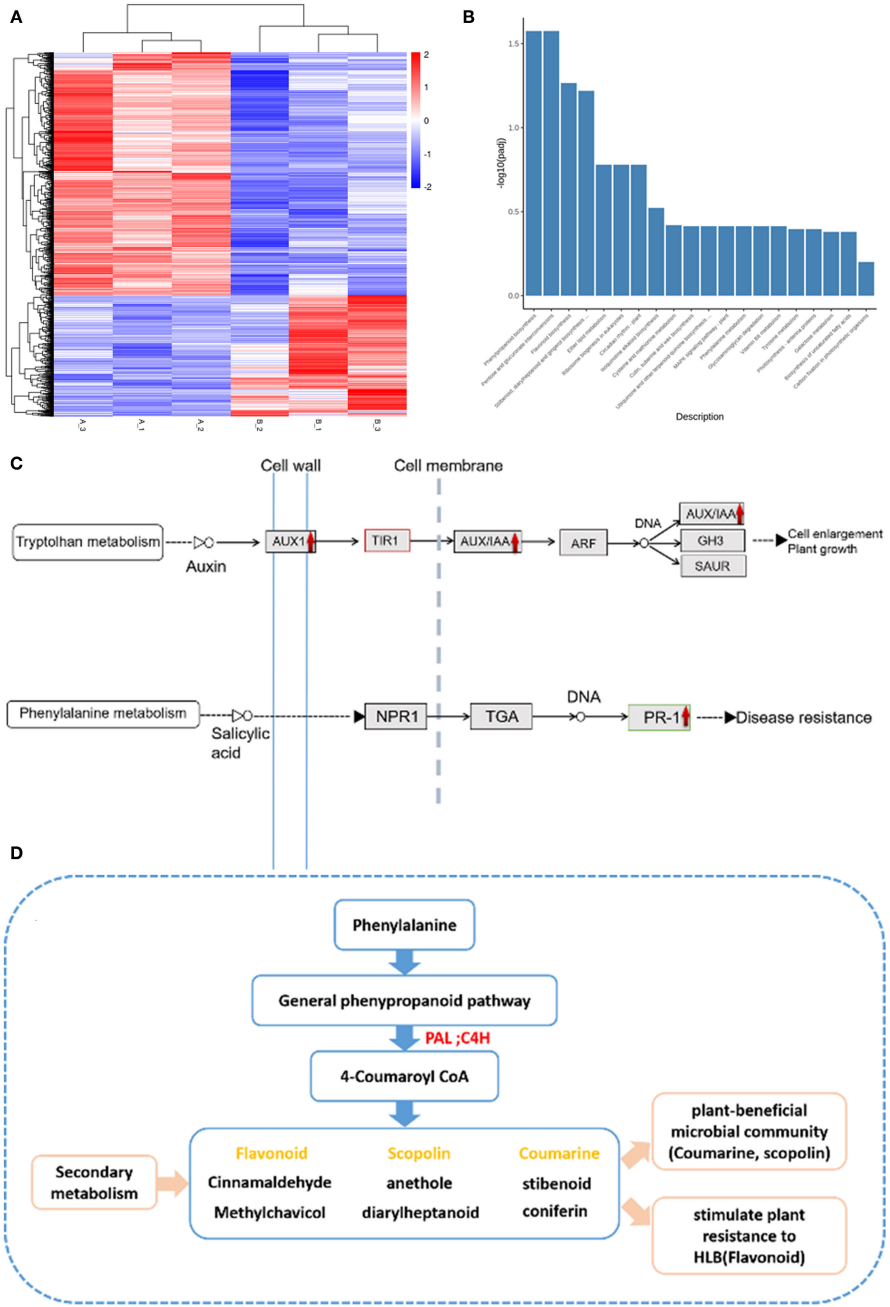
Transcriptome experiments data analysis. **(A)** Expression profiles of DEGs between pre-treatment and post-treatment. Heat map for cluster analysis of DEGs by K-means method. Red indicates up-regulated and blue indicates down-regulated, comparison is **(B)** vs. **(A)**. 1,050 genes were up-regulated, and 527 genes were down-regulated as a consequence of treatment. **(B)** The KEGG enrichment. The most significant 20 KEGG pathways were selected for the histogram. The abscissa is the KEGG pathway, and the ordinate is the significance level of pathway enrichment. The higher the value, the more significant. **(C)** The pathway of plant hormone signal transduction. The red indicates up-regulation after treatment. The vertical dotted line represents the cell membrane and the vertical solid line represents the cell wall. **(D)** The secondary metabolism of the phenylalanine-related pathway. The red arrow indicates up-regulation after treatment with FOS and CAC, and yellow characters represent secondary products of beneficial bacteria and resistance genes. PAL, phenylalanine ammonia-lyase; C4H, 4-hydroxylase.

The heat-map suggested that the genes before and after treatment had undergone significant changes (Figure 7A). These results indicated that the DEGs (differentially expressed genes) transcription levels were higher in post-treatment samples than that in the pre-treatment samples of the citrus tree (Figure 7A). One thousand fifty genes were up-regulated, and 527 genes were down-regulated as a consequence of treatment. This suggests that our treatment changed most DEGs in the root from down-regulated (blue color) to up-regulated expression (red color), and the original up-regulated expression gene (red color) became down-regulated expression (blue color) (Figure 7A).

Among the 103 metabolic pathways (Supplementary Data 2), two of them that had the biggest changes of DEGs i.e., phenylpropanoid biosynthesis, pentose and glucuronate interconversions (Figure 7B). To obtain a deeper understanding of these two metabolic pathways and plant hormone signal transduction, we counted the number of DEGs before and after treatment, respectively, (Supplementary Table 4). Out of 50 DEGs, 41 were up-regulated and 9 were down-regulated after treatment. Five down-regulated DEGs belong to PP2C (phosphoprotein phosphatases type-2C) that contributes to reduce stomatal closure and seed dormancy, and improves the roots cellular respiration (Fuchs et al., 2012; Krzywinska et al., 2016). Among the genes up-regulated after treatment, *AUX1* (involved in hormonal signaling and growth) and *PR1* (involved in plant defenses) (Figure 7C) and the phenylalanine pathway (originating phenylpropanoid secondary products, such as coumarin, flavonoid, scopoletin (Zhang et al., 2015) were observed (Figure 7D). Additionally, 12 genes in pentose and glucuronate interconversions are up-regulated after our treatment: among 7 DEGs, pectin esterase is involved in plant defense against pathogens (Lionetti et al., 2017), PGA-lase (pectate lyase) and galacturan 1,4-alpha-galacturonidase and polygalacturonase (Supplementary Table 4) are involved in catalyzing the cleavage of pectic acid to produce oligosaccharides (Babbar et al., 2016).

## Discussion

Our objectives are to kill *T. semipenetrans* and various pathogens (including *Ca*.Las in the roots) and to inhibit the growth of *Ca*.Las in citrus. According to published paper and our own experimental results, there are a large number of *T. semipenetrans* juveniles on severely HLB-infected citrus. We used FOS to prevent *T. semipenetrans* from further damaging root tissues. Because mechanical damage is the most important way for plant pathogens to invade plants, we hypothesize that root treatment is useful to ameliorate HLB disease symptoms. FOS is considered to play a very important role in controlling citrus slow decline disease caused by *T. semipenetrans* (Salahi Ardakani et al., 2014). While focusing on the prevention and control of citrus HLB, we also pay attention and effectively control the harm of citrus *T. semipenetrans*, because of its frequent co-occurrence with HLB (Song et al., 2016). Our result confirmed that more *T. semipenetrans* juveniles were detected in the roots with severe HLB citrus. At the same time, we have observed a large number of rotten roots (DREX) in the roots of HLB-infected citrus (whether it is a slight HLB citrus root or a severely infected HLB citrus root), and *Ca*.Las in DREX was high (Supplementary Figure 2). DREX and Non-DREX contains various pathogens, because our unmapped reads results (Supplementary Data 1, it is the unmapped reads in citrus database of the B and C samples, then follow the results obtained after the NR database in NCBI blast) suggest that there are other pathogens in pre-treatment samples of both DREX and Non-DREX. We chose to use CAC to kill pathogens through root drenches 3 days after FOS treatment because it is a broad spectrum fungicide with low toxicity to humans and animals and has the function of promoting the growth of crops (Li et al., 2016). We used FOS first as it could alleviate further mechanical damage, then we used CAC to kill various pathogens including HLB bacteria (*Ca*.Las) in the roots. We found that FOS and CAC could be used together to inhibit the growth of *Ca*.Las with more statistical significance. After a period of time, the root health status had improved, we observed the growth of new root, and *Ca*.Las were reduced significantly (Supplementary Figure 1 and Figure 3). If the root health status is improved, the above-ground leaf *Ca*.Las will slowly decrease.

**Figure 8:**
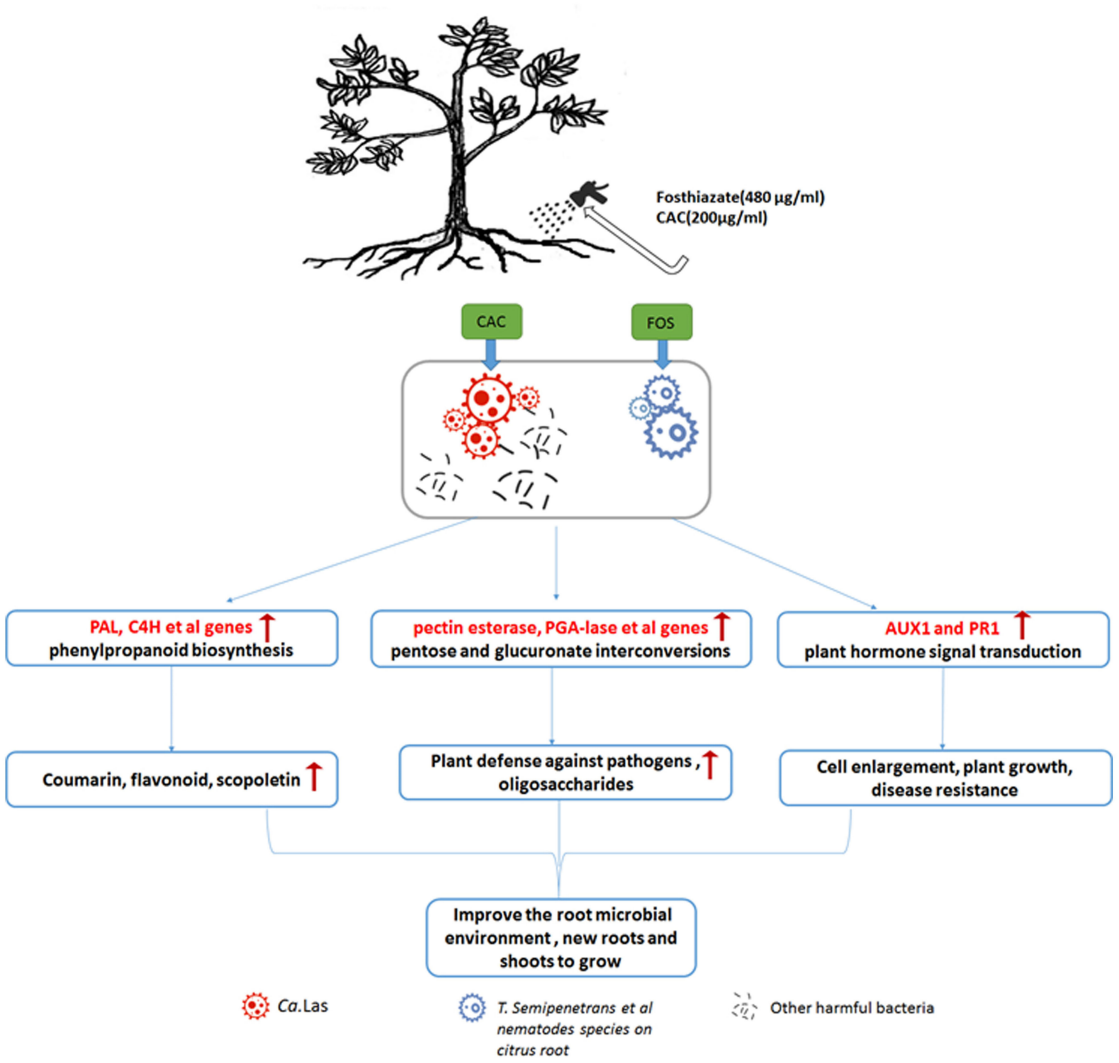
Schematic diagram of treatment method FOS and CAC combination through root drenches and a possible treatment mechanism. Our treatment plan is that for every sunny day, 480 μg/mL of FOS are provided to the HLB-infected citrus roots evenly through root drenches (pesticide FOS may eliminate *T. semipenetrans* and other nematodes on citrus root). After 3 days, the same roots are treated with 200 μg/mL of pesticide CAC (not only kill bacteria but also promote the greening of plant leaves) in a framework of correct orchard management. During this treatment, phenlypropanoid biosynthesis and pentose and glucuronate interconversion are promoted, resulting in the production of secondary metabolites, enhanced plant growth, induction of resistance, and differentiation of new roots and shoots. As a consequence, HLB-infected trees will improve their health condition, and the population of rhizosphere and endophytic pathogens will be controlled. In case of severe HLB infection, one more CAC treatment is envisaged. The treatment methods in greenhouse and the orchard are similar, but there is no need to worry about the weather, the treatment is carried out at any time, and the leaves are sprayed with CAC (same concentration as root drench).

Our treatment plan is that for every sunny day, add 480 μg/mL of FOS to the HLB-infected citrus roots evenly through root drenches (pesticide FOS may eliminate *T. semipenetrans* and other nematodes on citrus root), after 3 days, water the same roots with 200 μg/mL of pesticide CAC (not only to kill bacteria but also to promote the greening of plant leaves) and then followed by normal orchard management. During this treatment, the HLB-infected citrus tree will slowly regain its vitality, stay in strong health conditions, thus its own immune system will inhibit further HLB growth, and subsequently improve the root microbial environment, cause more new roots and shoots to grow (Figure 8). The above treatment options are suitable for moderate to slight HLB citrus trees. For citrus trees with severe HLB, CAC will consider use one more time. The treatment methods in greenhouse and the orchard are similar, but there is no need to worry about the weather, the treatment is carried out at any time, and the leaves are sprayed with CAC (same concentration as root drench). Interestingly, in greenhouse experiments, the HLB-infected potted citrus trees are produced by grafting, theoretically, there would not be many *T. semipenetrans* in the soil, but the treatment effect is more significant than in an orchard field infected by HLB (Supplementary Figure 1). This result is very surprising to us. Here we speculate that part of HLB disease symptomatology is due to root collapse, that other pests/pathogens attacking the root system (including nematodes) would worsen it, and root treatments aiming at reducing pest/pathogen population and stimulating the emission of new roots would improve plant health.

According to our antibacterial experiments and cell membrane permeation experiments, we show that FOS has a synergistic effect on CAC antimicrobial effect (Figures 4, 5), which is very interesting. FOS alone has a very low antibacterial rate, but together with CAC, it has a very good antibacterial effect. FOS ability together with CAC is more efficient antibacterial rate than CAC itself. The treatment program we set up is actually to administer FOS and CAC separately, and there is a 3–5 days' interval. Now it seems that the FOS and CAC work together to have a higher antimicrobial effect efficiency than what FOS or CAC treatment could accomplish alone. This interaction effect appears promising and will be the object of future investigation.

Starch accumulation is a common symptom of HLB in citrus. If there is a problem in the starch metabolism process, it will cause transitional starch accumulation in the leaves, destroy the thylakoid structure, directly hinder and affect photosynthesis (Goldschmidt and Huber, 1992), and cause leaf senescence. Our results show that starch accumulation is more severe in HLB citrus leaves, the swelling of starch granules destroys thylakoid structure in the chloroplast, and the secretory tissues are also blocked by starch and other substances (Figure 6). The PP2 gene (a phloem-specific lectin PP2-like protein) is considered to be a defensive response to prevent the pathogen from spreading further in the sieve tube, causing the sieve tube to block, leading to the accumulation of starch in the leaves (Albrecht and Bowman, 2008). The TUB8 genes (padj < 0.05), coding for β-tubulin, were up-regulated after treatment (Supplementary Data 4), which may contribute to the degradation of starch in citrus plants. In fact, the silencing of TUB8/β-tubulin 8 lead to leaf starch accumulation excess (Wang et al., 2015). Our transcriptome data showed a total of 35 PP2 genes, of which 26 gene expression was down-regulated after treatment (Supplementary Data 3), suggesting that PP2 down-regulation is beneficial to the reduction of starch accumulation. Studies have shown that starch accumulation and chlorophyll content have undergone a good change under the effective treatment (Pitino et al., 2020). Our results showed that FOS and CAC combination treatment reduced the starch accumulation (Figure 6D). Since our sampling time is all in the afternoon, we have observed that there is a lot of starch accumulation in healthy citrus leaves. It should be best to sample in the early morning when doing similar experiments in the future.

Our results show that the combined FOS + CAC therapy is indeed very effective. Based on our transcriptome results, most of the previously expressed genes have become low and most of the previously low expressed genes have become high before and after treatment, indicating that the fundamental metabolism has undergone drastic changes. This part of the data has yet to be developed and utilized. Based on the current transcriptome, we suggest that the secondary metabolites (coumarin, flavonoid et al.) of the phenylpropanoid biosynthesis pathway might become active during the treatment (Figure 7), which help the plant fights against HLB.

We carried out the determination of the physiological indicators of leaves' starch, total chlorophll content, disease resistance gene expression and roots vigor during FOS+CAC combination treatment. Compared with before treatment, these physiological indicators have been transformed to indicate better conditions after treatment: starch content decreased, chlorophyll content increased, disease resistance gene expression increased, and root vitality increased. We found that the combination method of FOS and CAC could effectively treat HLB-infected citrus roots and thus control the severity of citrus HLB disease, and that can be achieved with root treatments. After root drench treatment, *Ca*.Las in HLB citrus leaves were significantly reduced, and new roots grew, and root vigor was greatly enhanced; chlorotic leaves turned green, and their starch content was significantly reduced. In short, our combination treatment with FOS and CAC through root drench is simple and easy to do. For slight HLB-infected orchards, only one treatment plus good orchard management is required to cure HLB trees. For orchards with severe HLB infection, the number of treatments can be increased appropriately, and the orchard management must be strengthened at the same time to control the further spread of HLB and even cure HLB fruit trees.

When we conducted field experiments, we found that the HLB infection was actually accompanied by other citrus diseases, which means that the outbreak of Citrus HLB may be more likely to occur under chaotic conditions (all diseases occur at the same time). During our actual treatment process, we found that after one treatment, the orchard management could not keep up and the HLB symptoms could rebound after the initial decrease. The orchard management is also very important. Farmers need to apply more care and keep up with the spread or else the HLB will break out again. If the orchard is poorly managed after application, HLB cannot be effectively controlled. We even think that good orchard management, by itself, can control more than 50% of HLB spread. Our treatment with reasonable orchard management will have a significant healing effect.

Our results indicated that combining two drugs FOS and CAC effectively control citrus HLB. It prompts us to study the relationship between citrus *T. semipenetrans* and *Ca*.Las, which may be important for treating HLB and understanding the mechanism of HLB. In addition, improving the microbial environment of soil around citrus roots for promoting root growth may provide an important strategy to control HLB. Next, we will further study the effective mechanism of treatment and the convenience of environmental protection, such as the autophagy-related genes we recently studied and NPR1 to jointly regulate starch metabolism to make our own contributions.

## Data Availability Statement

The datasets presented in this study can be found in online repositories. The names of the repository/repositories and accession number(s) can be found in the article/Supplementary Material.

## Author Contributions

XL: data curation. WC, BC, and QZ: funding acquisition. JD, JZ, and SL: investigation. HL: methodology. WC and JD: writing–original draft. WC: writing–review and editing. All authors: contributed to the article and approved the submitted version.

## Conflict of Interest

The authors declare that the research was conducted in the absence of any commercial or financial relationships that could be construed as a potential conflict of interest.

## Abbreviations

HLB: Citrus Huanglongbing
*Ca*.Las: *Candidatus Liberibacter asiaticus*
FOS: Fosthiazate
CAC: Cupric-Ammonium Complex
DREX: Degraded Root tissue with Exposed Xylem
DEGs: Differentially expressed genes
WSC: Water soaked was used as control
KEGG: Kyoto Encyclopedia of Genes and Genomes.
