# Supplementary material for: Cocktail Therapy of Fosthiazate and Cupric-Ammoniun Complex for Citrus Huanglongbing
Source: Front Plant Sci. 2021 Mar 31;12:643971. doi: 10.3389/fpls.2021.643971 (PMC8044827; doi:10.3389/fpls.2021.643971)
Supplement: Supplementary file 1 [file Data_Sheet_1.docx]

Unmapped reads show that the NR test of all C samples are inconsistent

There have Citrus mold, fungus, Virus

details as follows:

==> B_2/B_2_blast.txt <==

species_name hit(percent)

Citrus clementina 1845/4299(42.92%)

Citrus sinensis 784/4299(18.24%)

Citrus unshiu 264/4299(6.14%)

Citrus trifoliata 51/4299(1.19%)

Anaeromyxobacter sp. RBG_16_69_14 48/4299(1.12%)

Olea europaea var. sylvestris 28/4299(0.65%)

Vitis vinifera 25/4299(0.58%)

Gossypium barbadense 23/4299(0.54%)

21/4299(0.49%)

==> B_3/B_3_blast.txt <==

species_name hit(percent)

Citrus clementina 1153/5404(21.34%)

Citrus sinensis 663/5404(12.27%)

Citrus unshiu 250/5404(4.63%)

Exophiala aquamarina CBS 119918 238/5404(4.4%)

Tulasnella calospora MUT 4182 146/5404(2.7%)

Phialophora americana 100/5404(1.85%)

Cladophialophora carrionii CBS 160.54 90/5404(1.67%)

Glomus cerebriforme 77/5404(1.42%)

Rhizophagus irregularis DAOM 181602=DAOM 197198 77/5404(1.42%)

==> C_1/C_1_blast.txt <==

species_name hit(percent)

Citrus yellow vein clearing virus 1791/6324(28.32%)

Halomonas gudaonensis 585/6324(9.25%)

Indian citrus ringspot virus 524/6324(8.29%)

Soehngenia saccharolytica 416/6324(6.58%)

Tissierella creatinini 316/6324(5.0%)

Citrus clementina 183/6324(2.89%)

Bacteria 166/6324(2.62%)

Citrus sinensis 116/6324(1.83%)

Anaeromyxobacter sp. RBG_16_69_14 108/6324(1.71%)

==> C_2/C_2_blast.txt <==

species_name hit(percent)

Psathyrella aberdarensis 497/4469(11.12%)

Rhagoletis zephyria 231/4469(5.17%)

Fusarium sp. AF-12 204/4469(4.56%)

Dermatophagoides pteronyssinus 151/4469(3.38%)

Madurella mycetomatis 88/4469(1.97%)

Fusarium sp. AF-8 72/4469(1.61%)

Coprinellus micaceus 65/4469(1.45%)

Sarcoptes scabiei 63/4469(1.41%)

Steinernema carpocapsae 56/4469(1.25%)

==> C_3/C_3_blast.txt <==

species_name hit(percent)

Fusarium sp. AF-12 127/4352(2.92%)

Steinernema carpocapsae 109/4352(2.5%)

Madurella mycetomatis 81/4352(1.86%)

Verruconis gallopava 62/4352(1.42%)

Panagrellus redivivus 60/4352(1.38%)

Toxocara canis 58/4352(1.33%)

Tulasnella calospora MUT 4182 48/4352(1.1%)

Fusarium sp. AF-8 47/4352(1.08%)

Aedes aegypti 45/4352(1.03%)
