# Supplementary material for: Cocktail Therapy of Fosthiazate and Cupric-Ammoniun Complex for Citrus Huanglongbing
Source: Front Plant Sci. 2021 Mar 31;12:643971. doi: 10.3389/fpls.2021.643971 (PMC8044827; doi:10.3389/fpls.2021.643971)
Supplement: Supplementary file 5 [file Data_Sheet_5.docx]

Supplementary Data

# Supplementary materials and methods

## Combination treatment of two drugs FOS and CAC in greenhouse

The citrus trees for the drug treatment in the greenhouse were divided into group A and group B, three trees per group. Group A was treated with two drugs FOS and CAC in combination, group B was treated with water as a control. The treatment method of group A is as follows: the roots of the citrus tree were first soaked in the Fosthiazate solution 480 µg/ml (2000×) for 1L to every citrus roots (75% fosthiazate from Hebei Sannong Agricultural Chemical Co, Ltd.). After 3-5 days, we used 1L 200 µg/ml CAC through root drenches (40 mg/ml CAC stock solution, 200×) to every citrus roots. Leaf samples were collected after post-treatment of 0, 15, 30, 60, 90 and 120 days to test Ca.Las content, and collect 1d, 2d, 4d, 7d, 14d leaves to test the expression of *PR1* and *PR2*.

## Gene expression analysis

Total RNA was extracted using Genomic RNA Geometry Extraction Kit of the Biotech Plant (Sangon, Shanghai, China) following the manufacturer’s instructions. The quantity of RNA samples was determined using a NanoDrop ND-1000 spectrophotometer (NanoDrop Technologies). The concentration of RNA samples was adjusted to 200 ng/µl as a template. cDNAs were obtained with Prime Script TM RT Master Mix Kit (Takara, Liaoning, China), and used as templates for real-time qPCR to express genes. All real-time qPCR reactions were repeated at least three times. GAPDH were used as internal control (Francis *et al.*, 2009). The primers used are listed in Supplementary Data Table 5.

## Determination of chlorophyll content

Chlorophyll contents were determined following Liu et al (Liu *et al.*, 2018). Leaves chlorophyll were extracted using 10mL 80% (V/V) acetone in the dark for 48h. The absorbance of supernatant was determined at 645 and 663 with spectrophotometer (UV-2550, Shimadzu, Japan). Total chlorophyll content was measured and normalized as previous described (Smith and Melis, 1987).

## Root vigor test

TTC (triphenyltetrazolium chloride) method is generally used to detect root vitality (Yamauchi *et al.*, 2014). TTC aqueous solution is colorless and can be reduced to produce TTF (triphenylmethylhydrazone), TTF is red, so the root activity of the sample can be qualitatively analyzed by the color change. According to extracting dehydrogenase from plant roots reduce TTC to TTF to reflect the strength of root vitality, we used Bestbio Root Vigor Detection Kit BB-52501 (http://www.bestbio.com.cn/goods.php?id=10231#detail) to carry out the root vigor experiment. Refer to the instructions for specific steps. The standard curve measured is：

$$\mathbf{y = 0.0357 x + 0.0142}$$

R^2^ = 0.9946,(y refers to A_485_, x refers to TTF concentration (μg/ml)).

$$\mathbf{Root (dehydrogenase) activity (mg/g.h) = m/(1000}\mathbf{W}\mathbf{t)}$$

(m: the amount of TTF of the sample extracted according to the standard curve, μg；W: weight of plant fibrous root system, g; t: incubation time, 1h).

# Supplementary Figures and Tables

## Supplementary Tables

**Table 1.** Materials: samples (Leaves midrib) are taken from 8-year-old *Citrus reticulata Blanco cv. Shatang Ju* with severe Huanglong disease in Xiaoshan village, Chonghua district, Guangzhou city.

| **Sample number** | **Ct value（Technology Repeat 1）** | **Ct value（Technology Repeat 2）** | **Ct value（Technology Repeat 3）** |
| --- | --- | --- | --- |
| Citrus 1 | 23.66 | 23.62 | 23.56 |
| Citrus 2 | 26.63 | 26.56 | 26.64 |
| Citrus 3 | 31.61 | 31.64 | 31.68 |
| Citrus 4 | 25.76 | 25.39 | 25.30 |
| Citrus 5 | 31.63 | 31.86 | 31.71 |
| Citrus 6 | 22.14 | 21.90 | 21.97 |
| Negative control | NA | NA | NA |

**Table 2.** Materials: samples (Leaves midrib) are taken from 8-year-old *Citrus reticulata Blanco cv. Shatang Ju* with slight Huanglong disease in Qiaotou village, Shuangjiang town, Dongyuan conunty, Heyuan city.

| **Sample number** | **Ct value（Technology Repeat 1）** | **Ct value（Technology Repeat 2）** | **Ct value（Technology Repeat 3）** |
| --- | --- | --- | --- |
| Citrus 1 | 37.49 | 37.31 | 37.52 |
| Citrus 2 | 35.88 | 34.27 | 35.94 |
| Citrus 3 | 38.66 | 38.61 | 38.75 |
| Citrus 4 | 38.48 | 38.42 | 38.59 |
| Citrus 5 | 37.21 | 37.32 | 37.26 |
| Citrus 6 | 37.49 | 37.53 | 37.66 |
| Citrus 7 | 34.94 | 35.11 | 35.05 |
| Citrus 8 | 38.56 | 38.63 | 38.71 |
| Citrus 9 | 39.00 | 38.94 | 38.83 |
| Citrus 10 | 37.21 | 37.33 | 37.41 |
| Negative control | NA | NA | NA |

**Table 3.** Summary of sequencing data from the different replicates of the pre-treatment transgenic line and post-treatment line.

| **Sample** | **Raw reads** | **Clean reads** | **Clean bases** | **Error rate** | **Q20** | **Q30** | **GC Content(%)** | **Total Mapped(%)** | **Uniquely Mapped(%)** |
| --- | --- | --- | --- | --- | --- | --- | --- | --- | --- |
| A_1 | 58701776 | 56250394 | 8.44G | 0.03 | 97.29 | 92.5 | 44.42 | 44710730(79.49%) | 43744386(77.77%) |
| A_2 | 56287268 | 54733602 | 8.21G | 0.03 | 97.22 | 92.38 | 44.45 | 43545235(79.56%) | 42580723(77.8%) |
| A_3 | 58720828 | 56528368 | 8.48G | 0.03 | 97.31 | 92.59 | 44.45 | 43482401(76.92%) | 42661786(75.47%) |
| B_1 | 53691664 | 52824002 | 7.92G | 0.03 | 97.47 | 92.88 | 44.16 | 40091156(75.9%) | 39028083(73.88%) |
| B_2 | 56871792 | 55747806 | 8.36G | 0.03 | 97.49 | 92.94 | 43.88 | 40425240(72.51%) | 39694121(71.2%) |
| B_3 | 54724178 | 53806158 | 8.07G | 0.03 | 97.54 | 92.97 | 44.65 | 45240571(84.08%) | 44083363(81.93%) |

^1^A_1/2/3 represent three replicates of post-treatment and group B_1/2/3 represent three replicates of pre-treatment

**Table 4.** The number of DEGs in different Pathways between A (post-treatment) and B (pre-treatment).

| **Pathway** | **Gene** | **No. change** | **A vs B** |
| --- | --- | --- | --- |
|  |  |  | **No. up/down** |
| Plant hormone signal transduction | *AUX1* | 2 | 2/0 |
|  | *SAUR* | 3 | 2/1 |
|  | *AUX/IAA* | 3 | 3/0 |
|  | *PYR/PYC* | 1 | 1/0 |
|  | *PP2C* | 5 | 0/5 |
|  | *BR11* | 1 | 1/0 |
|  | *CYCD3* | 1 | 1/0 |
|  | *PR-1* | 1 | 1/0 |
|  | *AHP* | 1 | 0/1 |
| Phenylpropanoid biosynthesis | *PAL* | 1 | 1/0 |
|  | *C4H* | 1 | 1/0 |
|  | *4CL* | 0 | 0/0 |
|  | *POD* | 11 | 11/0 |
|  | *shikimate O-hydroxycinnamoyl transferase* | 4 | 3/1 |
|  | *cinnamyl-alcohol dehydrogenase* | 1 | 1/0 |
|  | *coniferyl-alcohol glucosyltransferase* | 1 | 0/1 |
| Pentose and glucuronate interconversions | *Pectinescerase* | 7 | 7/0 |
|  | *PGA-lase* | 3 | 3/0 |
|  | *Pectinase* | 1 | 1/0 |
|  | *galacturan 1,4-alpha-galacturonidase* | 1 | 1/0 |

**Table 5** Target genes, their encoding protein and corresponding primers used in gene expression analysis by quantitative reverse transcription polymerase chain reaction**.**

| Target gene | Primer/probe sequence (5’–3’) | Function of encoding protein | Reference |
| --- | --- | --- | --- |
| *CsPR1* | Forward: AACTCGCCTCAAGACTACCT | Anti-fungal and anti-ooymycetic | (Dutt *et al.*, 2016) |
|  | Reverse: TGCAACTGTGTCGTTCCATA |  |  |
| *CsPR2* | Forward: TTCCACTGCCATCGAAACTG | β-1,3-Glucanase | (Francis *et al.*, 2009) |
|  | Reverse: TGTAATCTTGTTTAAATGAGC |  |  |
| *GAPDH* | Forward:  GGAAGGTCAAGATCGGAATCAA | GAPDH-C Citrus sinensis (Internal control) | (Francis *et al.*, 2009) |
|  | Reverse:  CGTCCCTCTGCAAGATGACTCT |  |  |

## Supplementary Figures


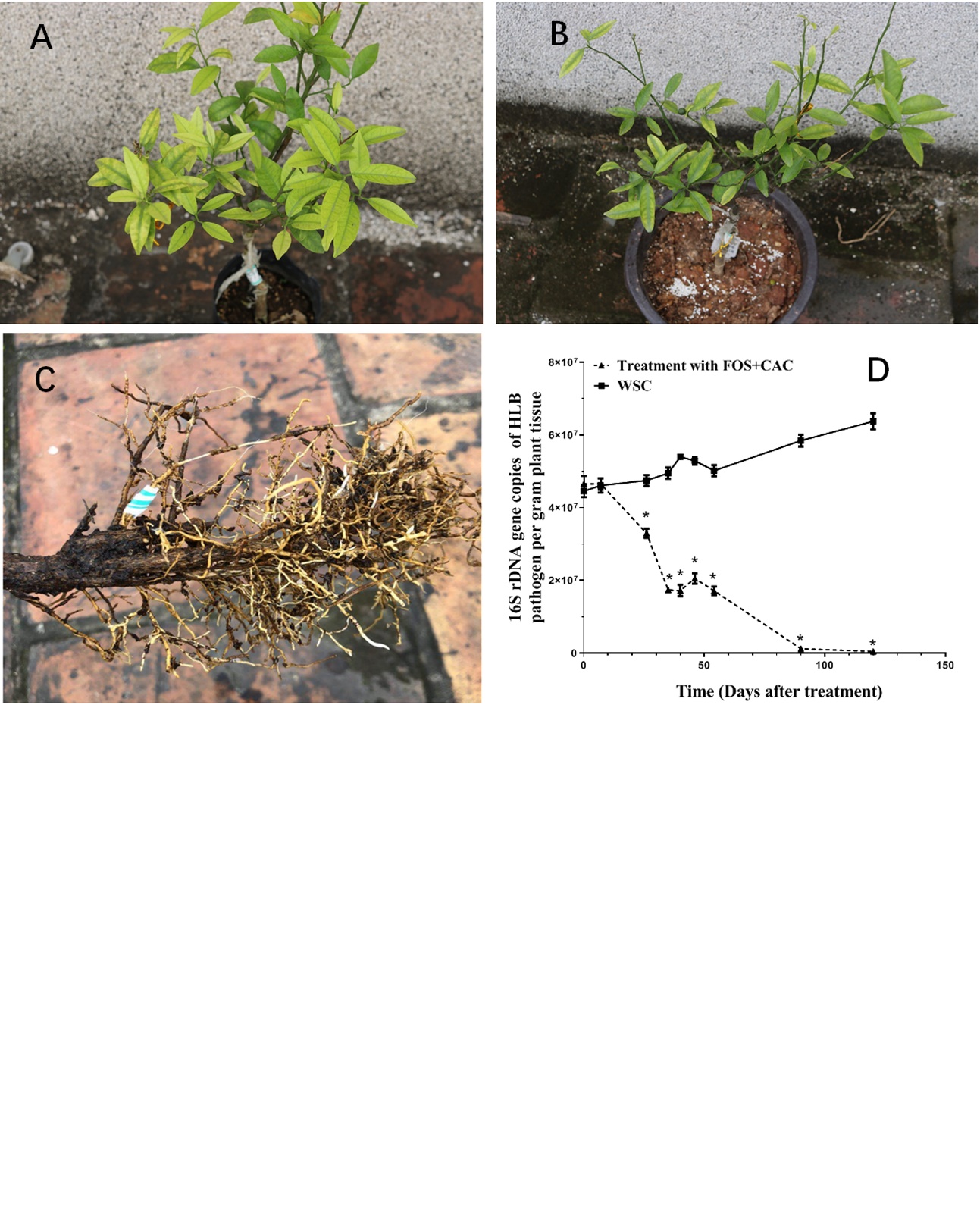


**S figure 1.** FOS and CAC combination effectively controlled HLB after treatment in greenhouse. **(A)**: The symptom of leaves before treatment. **(B)**: The symptom of leaves after 3 months treatment. **(C)**: The symptom of roots after 3 months treatment. **(D)**: 16S rDNA gene copies of *Ca*.Las per gram plant leaves midrib during 3 months in the orchard. Standard curve were obtained：Y=-0.310X+10.927，R^2^ = 0.9965,Y represents lg (16S rDNA gene copies of HLB pathogen)，X represents Ct value. WSC means water soak as control. An asterisk (*) indicates a significant difference (P<0.05; Dunnett’s test) between WSC (solid line) and FOS+CAC (dotted line).

**S figure 2.** HLB pathogen quantity comparison in a same HLB-infected citrus tree**.** HLB Pathogen in Degraded Root tissue with Exposed Xylem (DREX) is higher than Non-DREX and leaves.


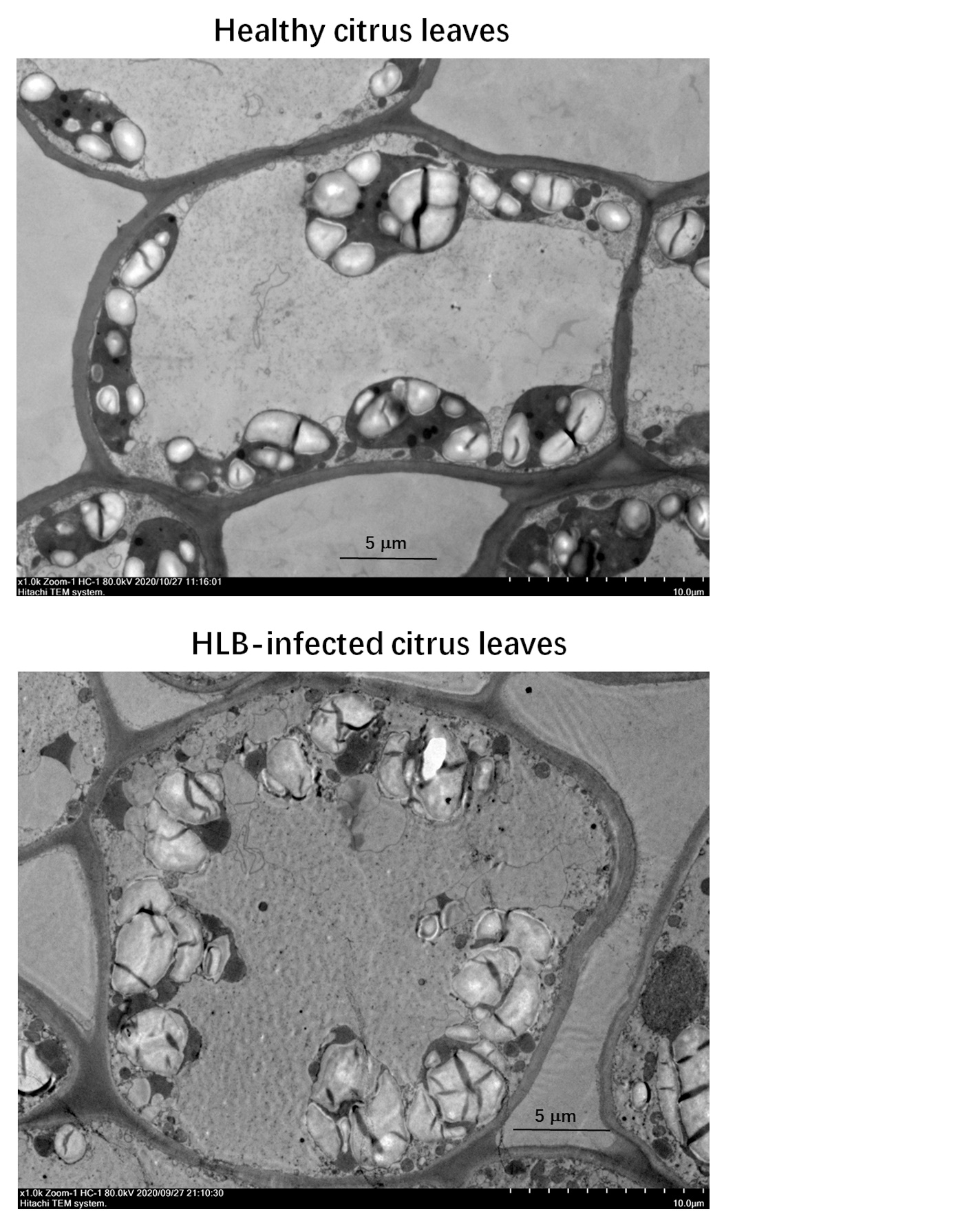


**S figure 3.** TEM images of citrus leaves: healthy citrus leaves; HLB-infected citrus leaves.


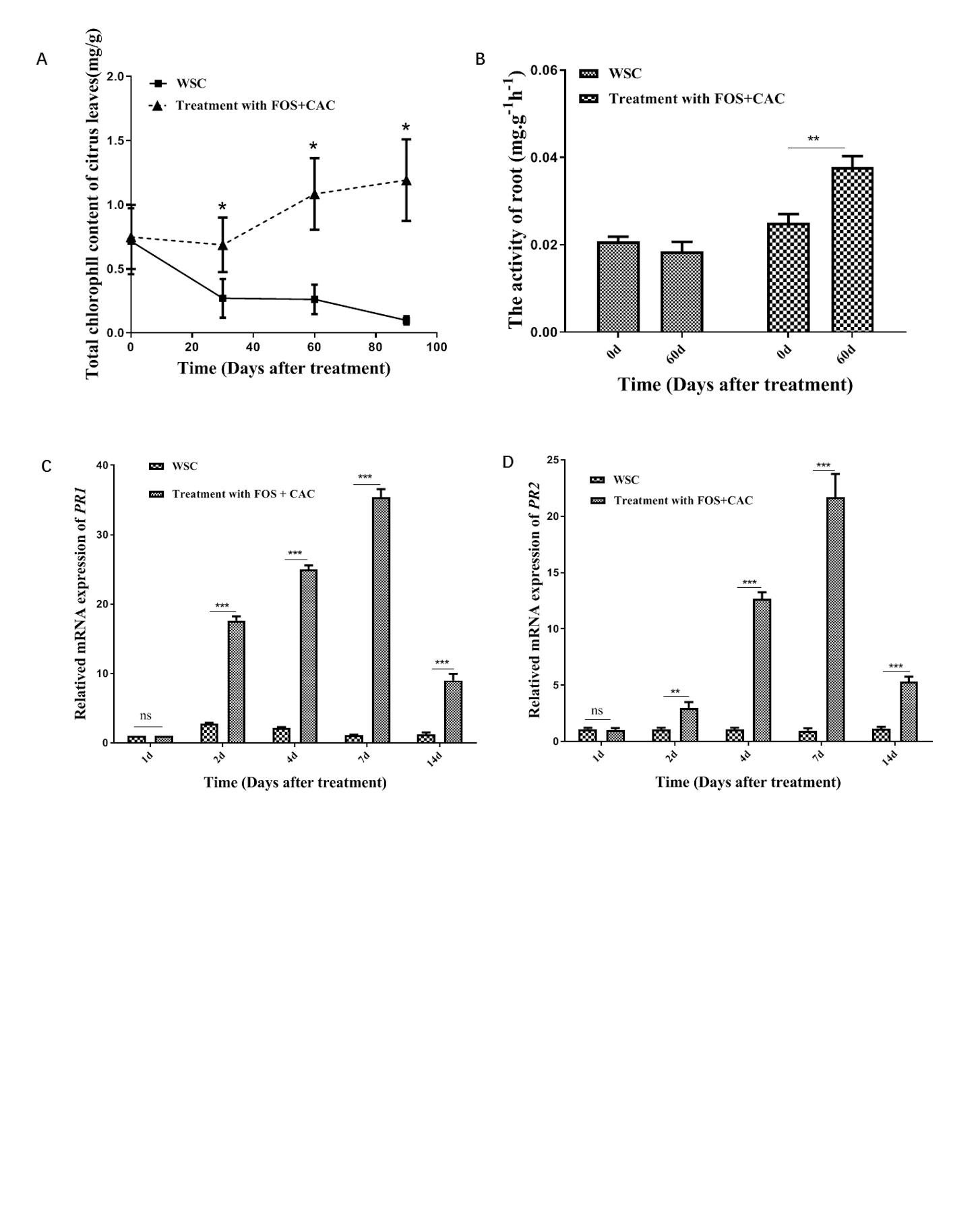


**S figure 4.** The changes of the total chlorophll content/*PR1*/*PR2* of HLB-affected citrus leaves and roots vigor during FOS+CAC combination treatment. **(A)**: The total leaf chlorophyll content in the orchard. An asterisk (*) indicates a significant difference (P<0.05; Dunnett’s test) between WSC and treatment with F+C. **(B)**: HLB-affected citrus roots vigor in the orchard. Asterisk (*) indicates significant differences between WSC and treatment with F+C. (Student’s t-test, **p < 0.01). **(C)**: *PR1* gene expression during F+C combination treatment 14 days in greenhouse. **(D)**: *PR2* gene expression during F+C combination treatment 14 days in greenhouse. The relative gene expression was calculated as fold change using the 2- ^ΔΔ^CT method. Each bar represents the mean of 3 replicates, with the error bars representing the standard deviation. Asterisk indicates significant differences in two groups (Student’s t-test, *p < 0.05, **p < 0.01, ***p < 0.001), ns indicated no significant differences in two groups.

**
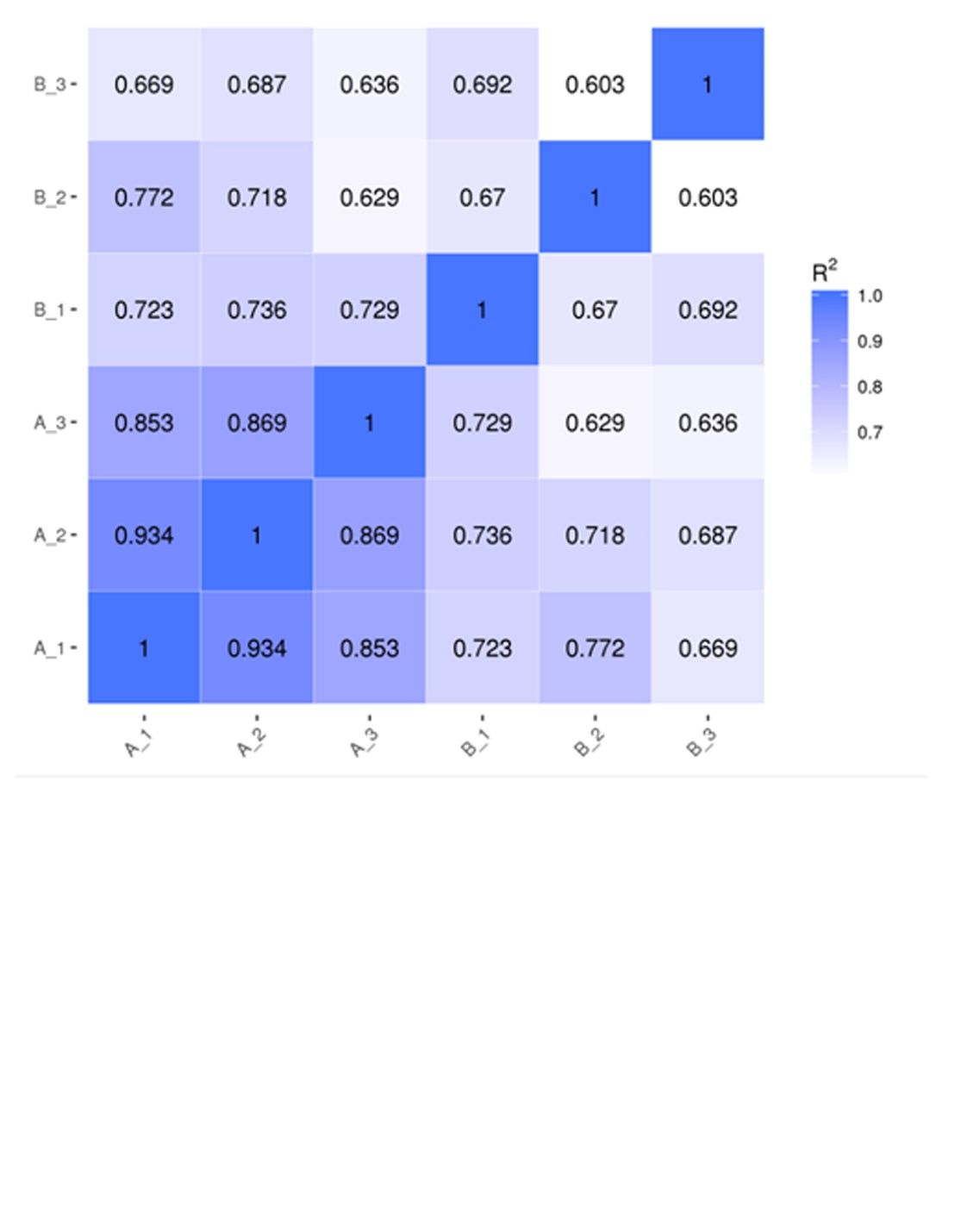
**

**S figure 5.** Transcriptome experiments data analysis. Correlation between RNA-Seq samples. A_1/2/3 represent three replicates of post-treatment and B_1/2/3 represent three replicates of pre-treatment R2 means the square of the Pearson coefficient.


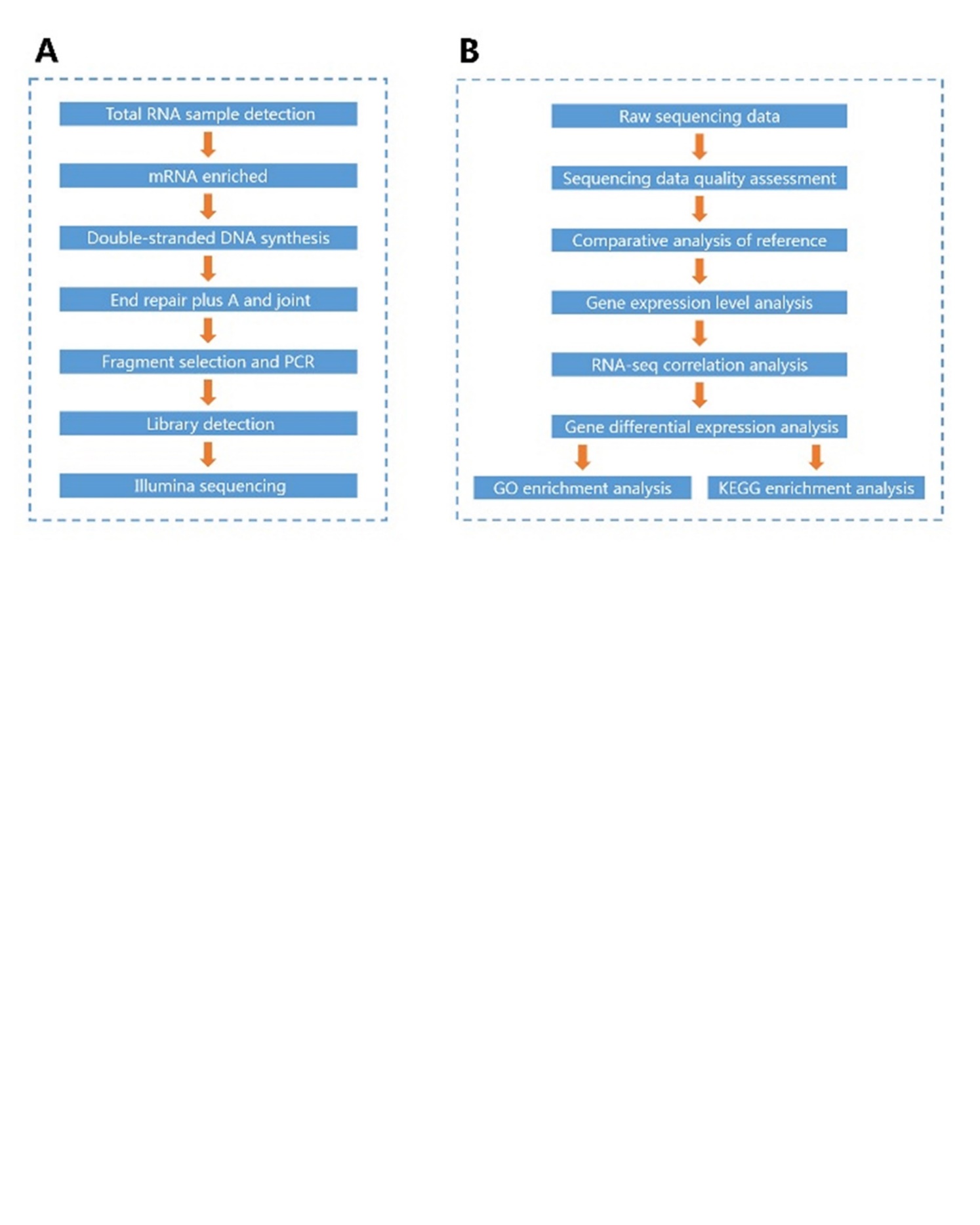


**S figure 6.** Procedure of RNA-seq data analysis. **(A)**: A flow chart of the transcriptome analysis. **(B)**: The information flow chart for the transcriptome analysis.
